# Methylene blue inhibits Caspase-6 activity, and reverses Caspase-6-induced cognitive impairment and neuroinflammation in aged mice

**DOI:** 10.1186/s40478-019-0856-6

**Published:** 2019-12-16

**Authors:** Libin Zhou, Joseph Flores, Anastasia Noël, Olivier Beauchet, P. Jesper Sjöström, Andrea C. LeBlanc

**Affiliations:** 1 Lady Davis Institute for Medical Research at Jewish General Hospital, 3999 Ch. Côte Ste-Catherine, Montreal, QC H3T 1E2 Canada; 2 Department of Anatomy and Cell Biology, McGill University, 3640 University Street Strathcona Anatomy Building, Montreal, QC H3A 0C7 Canada; 3 Department of Medicine, Division of Geriatric Medicine, Sir Mortimer B. Davis - Jewish General Hospital, 3999 Ch. Côte Ste-Catherine, Montreal, QC H3T 1E2 Canada; 4 Lee Kong Chian School of Medicine, Nanyang Technological University, Singapore, Singapore; 5 Centre for Research in Neuroscience, the BRaIN Program, Department of Neurology and Neurosurgery, McGill University, The Research Institute of the McGill University Health Centre, Montreal General Hospital, 1650 Cedar Avenue, Montreal, QC H3G 1A4 Canada; 6 Department of Neurology and Neurosurgery, McGill University, 845 Sherbrooke O, Montreal, QC H3A 0G4 Canada; 7 Bloomfield Center for Research in Aging, Lady Davis Institute for Medical Research, Sir Mortimer B Davis Jewish General Hospital, 3755 ch. Côte Ste-Catherine, Montréal, QC H3T 1E2 Canada

**Keywords:** Alzheimer disease, Axonal degeneration, Caspase-6, Caspase-6 inhibitor, Hippocampal CA1, Hippocampal fibres, Methylene blue, Synaptic plasticity, White matter

## Abstract

Activated Caspase-6 (Casp6) is associated with age-dependent cognitive impairment and Alzheimer disease (AD). Mice expressing human Caspase-6 in hippocampal CA1 neurons develop age-dependent cognitive deficits, neurodegeneration and neuroinflammation. This study assessed if methylene blue (MB), a phenothiazine that inhibits caspases, alters Caspase-6-induced neurodegeneration and cognitive impairment in mice. Aged cognitively impaired Casp6-overexpressing mice were treated with methylene blue in drinking water for 1 month. Methylene blue treatment did not alter Caspase-6 levels, assessed by RT-PCR, western blot and immunohistochemistry, but inhibited fluorescently-labelled Caspase-6 activity in acute brain slice intact neurons. Methylene blue treatment rescued Caspase-6-induced episodic and spatial memory deficits measured by novel object recognition and Barnes maze, respectively. Methylene blue improved synaptic function of hippocampal CA1 neurons since theta-burst long-term potentiation (LTP), measured by field excitatory postsynaptic potentials (f*EPSPs*) in acute brain slices, was successfully induced in the Schaffer collateral-CA1 pathway in methylene blue-treated, but not in vehicle-treated, Caspase-6 mice. Increased neuroinflammation, measured by ionized calcium binding adaptor molecule 1 (Iba1)-positive microglia numbers and subtypes, and glial fibrillary acidic protein (GFAP)-positive astrocytes, were decreased by methylene blue treatment. Therefore, methylene blue reverses Caspase-6-induced cognitive deficits by inhibiting Caspase-6, and Caspase-6-mediated neurodegeneration and neuroinflammation. Our results indicate that Caspase-6-mediated damage is reversible months after the onset of cognitive deficits and suggest that methylene blue could benefit Alzheimer disease patients by reversing Caspase-6-mediated cognitive decline.

## Introduction

AD is defined by the appearance of age-dependent progressive cognitive impairment, brain amyloid beta peptide (Aβ)-laden extracellular plaques, intraneuronal aggregated Tau protein-containing neurofibrillary tangles (NFT), synaptic loss, and neuroinflammation. There are currently no effective treatments to prevent or delay progressive memory loss and dementia of AD. One possible reason for the absence of an effective treatment against AD may be that current therapeutic strategies do not target the neurodegenerative events initiating cognitive deficits and pathological features. Thus, testing novel and earlier therapeutic targets may be critical for the identification of an efficient treatment against AD.

Caspase-6 (Casp6), a member of the cysteine-aspartic protease caspase family involved in apoptosis and inflammation, may represent an early therapeutic target of AD. Casp6 activation, detected with neoepitope antisera against the processed active subunit of Casp6 and Tau cleaved by Casp6 (Tau∆Casp6), is abundantly present in prototypical neuropil threads, neuritic plaques, and NFT of sporadic and familial AD [5, 6, 20]. Cerebrospinal fluid Tau∆Casp6 levels reflect brain levels of Tau∆Casp6 and predict lower cognitive performance in AD [40]. Cleavage of Tau protein by caspases is possibly associated with its aggregation in AD [13, 16, 20]. In aged non-cognitively impaired (NCI) individuals, active Casp6 is observed in entorhinal cortex (ERC) and hippocampal CA1 neurons [6, 20, 39], the first areas presenting NFT in AD [8]. In these individuals, higher levels of Casp6 activity correlate with lower performance in episodic and semantic memory, the two types of memory first affected in AD [39].

In cultured neurons, active Casp6 cleaves several cytoskeletal or cytoskeletal-associated proteins essential to normal neuronal function, some of which, such as Tau protein are involved in AD [26], and induces axonal degeneration [12, 32, 43, 44]. Furthermore, activation of Casp6 in human primary neurons increases Aβ production [24, 27, 28].

Transgenic expression of a self-activated form of human Casp6 in mouse hippocampal CA1 neurons is sufficient to cause age-dependent cognitive deficits, neuroinflammation, and neurodegeneration, in the absence of Aβ plaques and NFT [29]. Casp6-mediated neurodegeneration may be specific to hippocampal CA1 neurons since Casp6 expression and activation in the striatal medium spiny neurons fails to induce neurodegeneration and neuroinflammation, nor does it induce striatum-dependent behavioral abnormalities [33]. These data suggest that inhibition of Casp6 in the very early stages of AD may prevent early cognitive deficits and AD pathology.

A preliminary study indicated that the treatment of Casp6 transgenic mice with the NWL-117 caspase peptide inhibitor reverses cognitive deficits [35]. However, the NWL-117 or other Casp6 inhibitors are far from being sufficiently developed for use in human clinical trials. Recently, methylene blue (MB) has been shown to inhibit caspases by oxidizing their catalytic cysteinyl residue [36]. MB has several drug-like properties in terms of safety [31], absorption [11], blood-brain barrier permeability, and distribution in the CNS [37]. Pharmacokinetic studies showed that MB levels are 60-fold higher in brain tissue than in plasma and its half-life in brain is approximately 30 h [23]. MB reduces insoluble and phosphorylated Tau and prevents cognitive deficits in Tau transgenic mice [23, 34]. MB is currently studied in clinical trials against AD due to its function as a Tau aggregation inhibitor [55]. The MB derivative, LMTX, initially failed to show a benefit for AD in a phase 3 clinical trial [17]. However, a subsequent report indicated that LMTX had a significant beneficial effect on cognitive scores, brain atrophy and glucose uptake when taken as a monotherapy [54].

The goal of this study was to investigate whether MB could reverse Casp6-mediated cognitive deficits, neurodegeneration, and neuroinflammation in mice overexpressing human Casp6. Our results show that MB reversed Casp6-induced episodic and spatial memory impairment, when administered 3 to 4 months after the onset of cognitive deficits, which occur at 15 months of age [29]. Casp6 activity was inhibited by MB in mice brains and in acute hippocampal slices. MB normalized LTP induction in the hippocampal Schaffer collateral-CA1 pathway in acute brain slices of Casp6-expressing mice. Furthermore, MB reversed Casp6-mediated increased brain neuroinflammation. This study demonstrates that Casp6-induced cognitive deficits, neuritic degeneration, and neuroinflammation are reversible and implies that human Casp6-dependent cognitive decline in AD may be prevented with a Casp6 inhibitor.

## Materials and methods

### Aim, design and setting of the study

The aim of this study was to assess the reversibility of Casp6-mediated age-dependent cognitive deficits in vivo with MB treatment. MB is a non-toxic, blood brain barrier permeable inhibitor of caspases [36] and is already being tested in human clinical AD trials [17, 54]. The ACL Caspase-6 KI/Cre mice are cognitively impaired at 15 months of age [29]. MB was administered 3 months after the onset of cognitive deficits to assess reversibility of well-established cognitive defects. Cognitive status was assessed at 18 months, and again at 19 months of age, after 1 month of treatment. Additional treatment and cognitive assessments were planned every month thereafter until 24 months of age or until an effect was observed. Since reversibility of cognitive deficits was observed within 1 month of treatment, animals were sacrificed at 19 months of age and submitted to electrophysiology of acute hippocampal slices, immunohistochemistry of brain sections and biochemical analyses of brain tissues.

### Human Casp6 transgenic mice

C57BL/6 Casp6 transgenic knock-in ACL (type I) mice (KI/Cre) express a self-activated form of human Casp6 lacking the pro-domain under Cre/loxP recombination. A floxed STOP sequence between human Casp6 and the CMV immediate early enhancer/chicken β-actin promoter in the knock-in gene was excised by Ca++/calmodulin kinase IIα (CAMKIIα)-regulated Cre expression, allowing the expression of human Casp6 in the hippocampal CA1 pyramidal cells [29, 50]. Since the transgene was inserted in the hypoxanthine-guanine phosphoribosyl transferase locus of chromosome X (*HPRT*), only males were used in all experiments to avoid lionization effects. The wild type (WT/WT), KI transgenics without Cre (KI/WT), or Cre only (WT/Cre) mouse littermates were used as controls. Casp6 Knock-Out (KO) mice were obtained from Jackson Laboratories (Bar Harbour, ME, USA). The type II ACL/G mice resulted from CAMKIIα-Cre recombinase gene expression in testis and deletion of the STOP sequence of the floxed transgene, allowing male germ line transmission of the STOP-excised transgene and thus whole body expression of a transgene in the F2 progeny [10]. Treatments and behavioral tests were done blinded to the ACL mice type and genotype. All animal procedures followed the Canadian Council on animal care guidelines, which were approved by the McGill University Animal Care Committees. Mice were bred and maintained in the Goodman Cancer Research Centre Mouse Transgenic Facility at McGill University and transferred to the Lady Davis Institute for experiments. Mice were housed at 22 °C, in 55% humidity, and on a reversed 12 h light/dark cycle with ad libitum food and water.

### Oral administration of MB

Mice of 18 months of age were used for the study since ACL human Casp6 transgenic mice displayed episodic and spatial memory impairment at the age of 15 months [29] and 18 month-old mice are considered old according to Jackson Laboratories. Eighteen-month-old littermates from KI/Cre (ACL/G *n* = 27, ACL *n* = 17), KI/WT (ACL/G *n* = 8, ACL *n* = 23), WT/Cre (*n* = 38), and WT/WT (*n* = 40) were administered approximately 20 mg/kg/day 3,7-bis(Dimethylamino) phenazathionium chloride (MB), (Sigma-Aldrich, St. Louis, MO, USA) and 2 mM saccharin (Sigma), or 2 mM saccharin (vehicle) only in drinking water for 38 days (including the 8-day behavioral tests). During treatment, 1 vehicle-treated WT/WT mouse died, 2 MB-treated mice died (ACL/G KI/Cre and WT/WT). This was expected for aged mice. The dose was chosen based on a previously published paper [23], where MB was given orally to test its ability to prevent Tau aggregation. The oral administration of MB to C57Bl6 mice was shown to be well-tolerated and led to peak concentrations in plasma and brain within 2 h of treatment, and a half-life of 4.7 h in plasma and 30.6 h in brain [34]. Oral bioavailability was ~ 18% and much better than that delivered by i.v. treatment. MB was delivered in drinking water based on a 1 mL/10 g water consumption rate of an adult mouse ad libitum, and actual water consumption rate was measured for each cage (1–3 mice per cage) every day to confirm that the mice drank the drug. MB-treated mice (body weight = 44.43 ± 1.48 g) consumed 4.25 ± 0.21 mL/day, and vehicle-treated mice (body weight = 44.95 ± 1.46 g) consumed 4.51 ± 0.13 mL/day. The body weights of animals were measured every week and did not change over the time of treatment.

### Behavior tests

After habituation to the experimenter for 1–2 weeks, KI/Cre (ACL/G *n* = 18, ACL *n* = 8), KI/WT (ACL/G *n* = 8, ACL *n* = 14), WT/Cre (*n* = 27), and WT/WT (*n* = 24) mice were sequentially tested in open field, novel object recognition, and Barnes maze tasks before and after treatment. Equipment was wiped with 70% ethanol to eliminate odour cues between each mouse.

#### Open field test

Each mouse was placed in a corner of a 40 cm × 40 cm × 30 cm cuboid acrylic plexiglass box, and was allowed to freely explore for 5 min. The HVS 2100 automated video tracking system (HVS Image, Hamptom, UK) divided the field in 4 rows × 4 columns yielding 16 virtual zones and analyzed the total distance travelled, the percentage of time moving, the number of entries into virtual zones, the time spent in each virtual zones, and the percentage of used zones.

#### Novel object recognition (NOR)

NOR task was performed 24 h after the open field test. Two identical objects were placed in the northwest and northeast corner at 5 cm from the walls and separated by 30 cm. Mice were placed in the box, where they encountered both objects. After 5 min, mice were replaced in their homecage. After 2 h, mice were returned to the NOR box for 5 min where a familiar object was replaced with a novel object. The position of the novel object was changed between each animal to avoid any bias related to a preference in the location of the new object and the use of possible confounding spatial cues. In addition to the tracking record by the HVS 2100 system, the experimenter recorded the number of times mice touched each object. Two WT/WT mice (vehicle- and MB-treated) and three ACL/G KI/Cre (1 vehicle- and 2 MB-treated) were excluded because they froze during the test. The discrimination index was calculated as the (number of time touching the novel object – number of time touching the familiar object)/(number of time touching both object).

#### Barnes maze

The Barnes maze apparatus consisted of a round 90 cm diameter table with 20 equidistant 5 cm-diameter holes on the edge. All the holes were blocked except for the target hole, which had an escape hatch under the table. The four walls around the table were pasted with high contrast visual cues to allow spatial memory. Mice were placed on the center of the table under a black box. When the trial started, the black box was taken away, and a strong light and buzzer served as the stimuli for mice to search for an escape. Once mice escaped successfully, the light and buzzer were turned off. The Barnes maze task was administered in three phases: adaptation (day 0), spatial acquisition training (day 1–4) and probe test (day 5). On the day of adaptation, mice were allowed to explore the table for 60 s, and stay in the hatch for 120 s. On days 1–4 of training for spatial acquisition, the escape latency and the number of errors made before finding the escape hatch were recorded. If the mouse could not escape within 180 s, the experimenter gently led it into the escape hatch and allowed it to remain there for 60 s. Mice were given four trials per day with an inter-trial interval of 15 min. On day 5, mice were given a single probe test, in which the target hole was blocked. The mouse was allowed to explore for 90 s, and the primary latency and primary errors to reach the escape hole, and the number of visits to each hole were recorded. The HVS 2100 automated video software tracked the animals during all trials. Two WT/Cre (vehicle- and MB-treated) and one MB-treated ACL KI/Cre were excluded because they froze during the test.

### Immunohistochemistry

After behavioral analyses, mice were anaesthetized by isoflurane (Thermo Fisher Scientific, Waltham, MA, USA), and perfused transcardially with 50 ml ice cold 0.9% saline and 200 ml 4% paraformaldehyde in 0.2 M Phosphate Buffer using a peristaltic pump (Thermo Fisher Scientific). Brains were removed and post-fixed in 10% formalin in Phosphate Buffer (Thermo Fisher Scientific) for 24 h and then dehydrated in 70% ethanol for 24 h. Fixed brains were paraffin embedded and cut with a microtome at the Institute for Research in Immunology and Cancer histology centre (University de Montreal, Montreal, Canada). Brains were serially sectioned at 4 μm through the anterior hippocampus between bregma − 1.22 μm and bregma − 1.52 μm. In total, 75 consecutive sections (3 sequential sections per slide) were collected to obtain 25 slides per mouse brain. Every fifth slide, corresponding to an interval of 60 μm, were used for immunohistochemical analysis of microglial Iba1, astrocyte GFAP, and Casp6 activity TubΔCasp6 (EEVGVD^438^) immunomarkers. The last slide was used for human Casp6 analysis. After deparaffinization in 100% xylene (Thermo Fisher Scientific) twice for 5 min each and rehydration in 100% ethanol twice for 5 min each, 95% ethanol once for 5 min, and MilliQ water for 5 min, slides were incubated in antigen retrieval buffer (10 mM Tris-base, 1 mM EDTA, 0.5% Tween-20, pH 9 for Iba1, human Casp6, and TubΔCasp6 and 0.01 M Tris-Na citrate, pH 6 for GFAP) at 97 °C for 20 min in the Pascal Dako Cytomation (Dako, Burlington, ON, Canada). Immunohistochemical staining was automatically performed by the automated Dako Autostainer Plus slide processor using the EnVision Flex system (Dako). Briefly, slides were treated with 0.03% hydrogen peroxide for 15 min, washed 3 times with Wash Buffer (Dako), blocked with Serum-Free Protein Block (Dako) for 30 min, and incubated with either 1/2000 anti-Iba1 (019-19741Wako, Richmond, VA, USA), 1/8000 anti-GFAP (z-0034 Dako), 1/5000 anti-TubΔCasp6 (GN20622, Laboratory made [26]), or 1/5000 anti-human Casp6 (LS-B477 Lifespan Bioscience, Seattle, USA), diluted in EnVision Flex Antibody Diluent (Dako) for 30 min. After rinsing in Wash Buffer, brain sections were incubated with anti-rabbit-HRP or anti-mouse-HRP for 30 min. Staining was visualized with 3,3′-diaminobenzidine substrate-chromogen for 10 min and counterstained with hematoxylin (Dako) for 5 min. After dehydrating the slides in MilliQ water for 5 min, 95% ethanol for 3 min, 100% ethanol twice for 5 min each, 100% xylene twice for 3 min each, slides were mounted with Permount mounting medium (Thermo Fisher Scientific).

Slides were scanned using the Mirax Scan (Zeiss, Germany). Three to five micrographs at 40x magnification were taken in the stratum oriens, pyramidal cell layer, stratum radiatum and stratum lacunosum molecular of each hippocampal CA1 region, corpus callosum, fimbria and fornix. Image J software was used to measure the positive immunoreactive area over the total area in square microns. The number of Iba1-positive subtype I-IV microglia were counted in each region by the experimenter blinded to genotype and treatments based on semi quantitative scoring schemes [4].

### Excision of the STOP sequence with CaMKII-α Cre recombinase

Genomic DNA from hippocampus or tail was extracted as described [7]. Twenty ng of genomic DNA was used as template to amplify the recombined or Cre-excised recombined HPRT locus using primers designed by Genoway: Forward 5′-TGCTTCAGTCCCATGTTTGGCAAGG-3′ (GX5889) and Reverse 5′-AAATCTGTGCGGAGCCGAAATCTGG-3′ (GX2752). The recombined HPRT locus and Cre-excised recombined HPRT locus generate a 4195 and 2812 bp amplicon, respectively. The amplicon was achieved using Q5 DNA polymerase with 35 cycles of 98 °C for 10 s, 61 °C for 30 s, 72 °C for 150 s, followed by a 72 °C extension period of 2 min.

Twenty ng of genomic DNA was used to amplify the CaMKII-α Cre using primers: Forward 5′- GACTAAGTTTGTTCGCATCCC − 3′ and Reverse 5′ – ATCCAGGTTACGGATAT AGT-3′. The ~ 1650 bp amplicon was achieved using Q5 DNA polymerase with 35 cycles of 95 °C for 30 s, 45 °C for 30 s, 72 °C for 60 s, followed by a 72 °C extension period of 10 min.

### RT-PCR and quantitative PCR (qRT-PCR)

Total RNA was extracted from mouse hippocampus, cortex and cerebellum with the miRNeasy Mini Kit (Qiagen, QC, CA) according to the manufacturer’s protocol. During RNA purification, genomic DNA was digested with the RNase-Free DNase in this kit. One μg of total RNA was converted to cDNA with the avian myeloblastosis reverse transcriptase (Roche, Mannheim, Germany). PCR amplification of a 229 bp human Casp6 amplicon was achieved with Taq DNA polymerase (New England Biolabs, Whitby, ON, Canada) and the 5′-CGATGTGCCAGTCATTCCTT-3′ and 5′-CTCTAAGGAGGAGCCATAT-3′ primers, 30 cycles of 95 °C for 30 s, 61.1 °C for 30 s, 68 °C for 60 s, followed by a 68 °C extension period of 10 min. The 904 bp murine Casp6 amplicon was amplified with 5′- CTCAGGGCTAGGACACCGGTGGGA-3′ and 5′- ATATATGTAGCAAGACAGATGGCC-3′ primers, 35 cycles of 95 °C for 30 s, 64 °C for 1 min, 68 °C for 1 min 30 s, followed by a 68 °C extension period of 5 min. The 151 bp 18S amplicon was obtained with the 5′-GTAACCCGTTGAACCCCAT-3′ and 5′-CCATCCAATCGGTAGTAGCG-3′ primers, 27 cycles of 95 °C for 30 s, 58 °C for 30 s, 68 °C for 60 s, followed by a 68 °C extension period for 5 min.

Quantitative RT-PCR was performed with SYBR Green Taq Mastermix (Quanta BioSciences, Gaithersburg, MD, USA) on the Applied Biosystems 7500 Fast Real-Time PCR apparatus (Applied Biosystems, Foster City, CA, USA). Murine Casp6 cDNA was detected with 5′-CATGCAGAAACCGATGGCTTCTA-3′ and 5′-GGACGCAGCATCCACCTGGGTCAC-3′ primers. 18S cDNA was amplified with 5′- GTAACCCGTTGAACCCCAT-3′ and 5′-CCATCCAATCGGTAGTAGCG-3′ primers. Results are expressed as fold-induction values normalized to the 18S reference gene using Pfaffl’s method [38].

### Western blot

Mouse hippocampus, cortex and cerebellum were dissected and frozen at − 80 °C until use. Proteins were extracted by homogenizing (TH Omni International, Marietta, GA) the tissue in 5 volumes of Tris-Triton lysis buffer (10 mM Tris pH 7.4, 100 mM NaCl, 0.1% SDS, 1 mM EDTA, 1% Triton X-100, and10% glycerol with freshly added 0.5% sodium deoxycholate, 300 μM 4-(2-aminoethyl)benzenesulfonyl fluoride hydrochloride, 2.4 μM pepstatin A, 2 μM leupeptin, 0.8 μM tosyl-L-lysyl-chloromethane hydrochloride, 1 mM phenylmethylsulfonyl fluoride, 1 mM sodium fluoride, and 1 mM sodium orthovanadate). After centrifugation at 13000 x g for 20 min, the supernatant protein concentration was quantified by Bradford assay (Bio-Rad, Canada). Five to twenty μg of protein were separated on 12% or 15% SDS-PAGE (7.5% for Tubulin) and transferred to PVDF membranes (Bio-Rad). Membranes were probed with either 1/1000 anti-mouse and human Casp6 (#9762, Cell Signalling Tech, USA), 1/5000 anti-human Casp6 (LS-B477, Lifespan Bioscience), 1/3000 anti-GFAP (z0334 Dako), or 1/5000 β-Actin (A5441, Sigma) in 5% non-fat milk in TBST (0.1 M Tris HCl, 1.5 M NaCl, 0.5% Tween-20) overnight at 4 °C. Incubation of horseradish peroxidase-linked anti-rabbit secondary antibodies (Jackson Immunoresearch Laboratories, West Grove, PA) at room temperature for 1 h were followed with enhanced chemiluminescence (GE Healthcare Life Sciences, QC, CA). Light emission from the immunopositive protein bands was visualized on X-ray films (Kodak, Rochester, NY, USA). Alkaline phosphatase-linked anti-mouse secondary antibody was used with NBT/BCIP substrate (Promega, WI, USA) for chromogenic detection of β-actin after the chemiluminescent detection. Quantifications were performed with the ImageJ software (NIH, Bethesda, MD, USA).

### Extracellular field recordings

After anaesthesia with isoflurane, 19-month-old vehicle-treated KI/Cre (ACL/G *n* = 4, ACL *n* = 1), ACL KI/WT (*n* = 6), WT/Cre (*n* = 6), and WT/WT (*n* = 6), and MB-treated KI/Cre (ACL/G *n* = 3, ACL *n* = 3), ACL KI/WT (*n* = 3), WT/Cre (*n* = 5), and WT/WT (*n* = 5) mice were perfused transcardially with 5 mL ice-cold N-Methyl-D-glucamine (NMDG, Sigma) artificial cerebrospinal fluid (ACSF; 93 mM NMDG, 2.5 mM KCl, 1.2 mM NaH_2_PO_4_, 30 mM NaHCO_3_, 20 mM HEPES, 5 mM Na-ascorbate, 3 mM Na-pyruvate, 12 mM N-Acetyl-L-cysteine, 10 mM MgCl_2_, 0.5 mM CaCl_2_, 25 mM glucose, pH 7.2, ~ 338 mOsm) saturated with 5% CO_2_/95% O_2_ [48]. Brains were removed quickly, submerged in ice-cold NMDG-ACSF, and cut into transverse 300 μm hippocampal slices with a 5000mz-2 vibratome (Campden Instruments Ltd., England). Brain slices were initially recovered in 32 °C NMDG-ACSF for 10 min, transferred to 32 °C normal ACSF (125 mM NaCl, 2.5 mM KCl, 1.25 mM NaH_2_PO_4_, 26 mM NaHCO_3_, 1 mM MgCl_2_, 2 mM CaCl_2_, 45 mM glucose, ~ 338 mOsm) for 20 min, and then placed in normal ACSF under 5% CO_2_/95% O_2_ for 30 min at room temperature.

All experiments were performed at 31–33 °C in normal ACSF saturated with 5% CO_2_/95% O_2_. The recording pipettes of 2–4 MΩ resistance were filled with normal ACSF and placed in the CA1 stratum radiatum. The stimulation electrode was also placed in the CA1 stratum radiatum 200 μm away from the recording pipette. Field recordings were boosted at 100x gain using a BVC-700A amplifier (Dagan Corporation, Minneapolis, MN) with low-pass filtering set to 5 kHz, and then recorded at 10 kHz with a PCI-6229 digitization board (National Instruments, Austin, TX) using custom scripts in Igor Pro 7 (WaveMetrics Inc., Lake Oswego, OR) running on a SuperLogics computer (Natick, MA). For baseline responses, Schaffer collaterals were recruited with 100-μs-long biphasic constant-voltage pulses, repeated once every 20 s, using a BSI-950 stimulus isolation unit (Dagan Corporation, Minneapolis, MN). Minimal and maximal stimulation levels were first determined in the 10 to 50 V range. Schaffer collaterals were then activated by a fixed intermediate voltage for the remainder of the experiment. LTP induction was achieved using a theta-burst protocol consisting of 12 trains of 4 pulses at 100 Hz delivered at 5 Hz and repeated three times at 0.1 Hz [1].

Recordings with unstable baseline were rejected, as assessed using a t-test of Pearson’s r [2]. Off-line analysis was performed using in-house software [2] running in Igor Pro 7 (WaveMetrics Inc., Lake Oswego, OR, USA). The slope of fEPSPs was normalized to the initial 20-min-long baseline period before averaging across recordings. Three or four LTP experiments were carried out in each animal.

### Neuronal Casp6 activity assay by FLICA

Acute brain slices from 19-month-old mice were obtained as described in the “Extracellular field recordings section. Brain slices were incubated with 1x Casp6-FLICA (FAM-VEID-FMK, ImmunoChemistry Tec., CA) in normal ACSF for 1 h at room temperature with 5% CO_2_/95% O_2,_ and rinsed three times in fresh ACSF for 10 min. We tested 3–4 slices for each animal for vehicle-treated KI/Cre (ACL/G *n* = 1, ACL *n* = 2), MB-treated KI/Cre (ACL *n* = 3), or vehicle-treated WT/WT (*n* = 3) mice, and 1 slice for each animal treated with z-VEID-FMK-treated KI/Cre (ACL/G *n* = 1, ACL *n* = 2) mice.

Two-photon excitation was achieved using a Chameleon XR Ti:Sa laser (Coherent, Santa Clara, CA, USA) tuned to 820 nm for carboxyfluorescein (FAM). The two-photon microscope was custom-built as previously described [1]. Imaging data were acquired using ScanImage v3.7 running in Matlab (The MathWorks, Natick, MA, USA) via PCI-6110 boards (National Instruments). Images of hippocampal CA1 regions at depths between 0 and 80 μm from the brain slice surface were taken from maximum intensity projections of two-photon laser scanning microscopy image stacks. Imaging areas were scanned at a rate of 2 ms/line and a resolution of 512 × 512 pixels with a slice separation of 2 μm. Each slice was an average of three green-channel frames. FLICA-positive neurons in CA1 regions were quantified at depths of 0, 10, 20 and 30 μm from the brain slice surface.

### Statistics

Statistical analyses of data were performed using Igor Pro 7 or GraphPad prism 7 (GraphPad Software, CA, USA). Comparison between two groups was done using the unpaired Student’s two-tailed t-test. Analysis comparing more than two groups with equal variance was done by one-way ANOVA. Brown-Forsythe’s ANOVA was used if Bartlett’s test indicated heteroscedasticity at the *p* < 0.05 level. Tukey’s post-hoc or Dunnett’s post-hoc test as indicated in figure legends was used when ANOVA indicated *p* < 0.05. Analysis of three or more groups at different time points (Barnes maze spatial acquisition training) was done using repeated-measures two-way ANOVA with Dunnett’s post-hoc test.

## Results

### Validation of human Caspase-6 gene expression in the KI/Cre mice

**Fig. 1:**
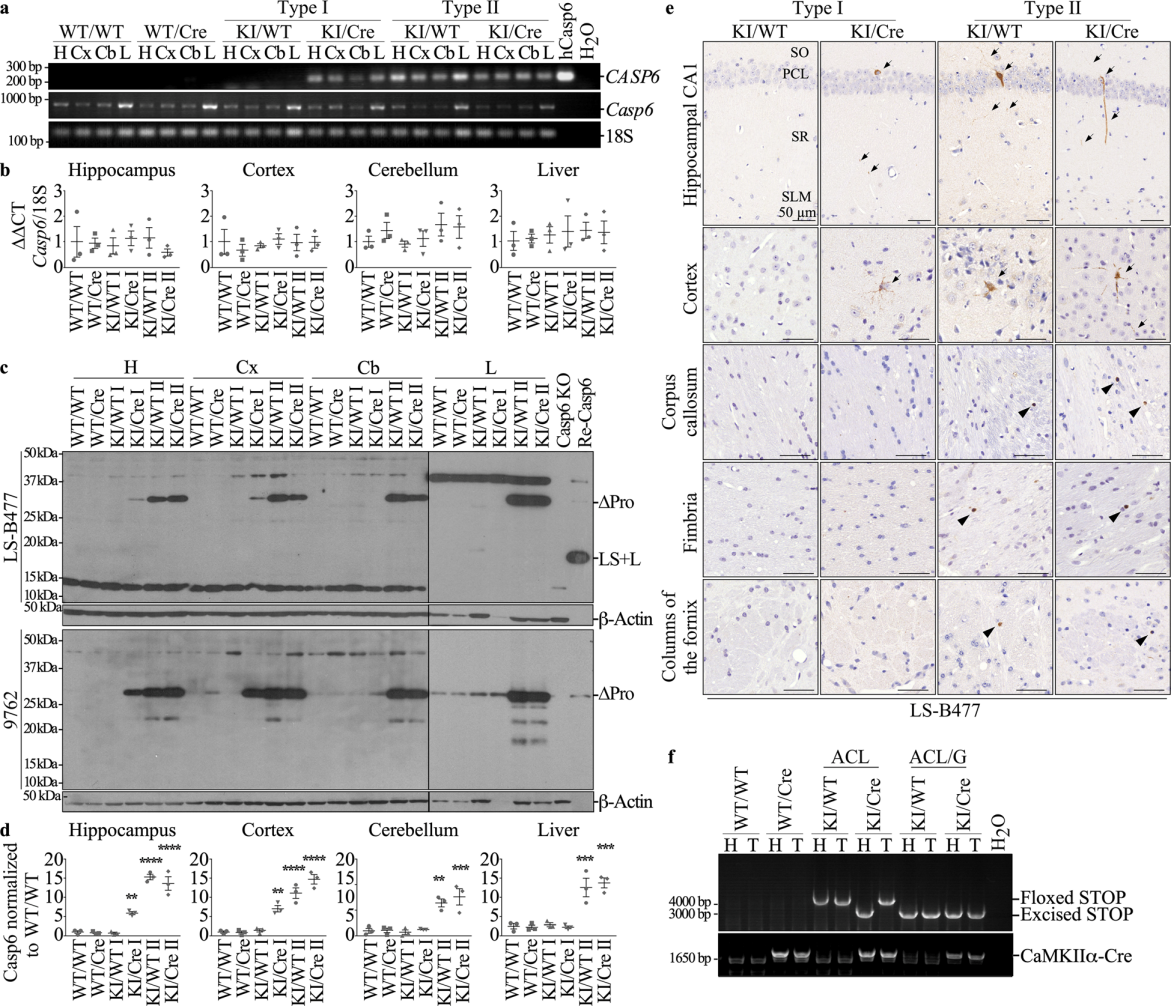
Human Casp6 expression in ACL and ACL/G mice. **a** Red safe-stained agarose gel of human *CASP6* (*CASP6*), murine *Casp6* (*Casp6*), or 18S RNA amplicons from hippocampus (H), cortex (Cx), cerebellum (Cb), or liver (L) of WT/WT, WT/Cre, Type I KI/WT and KI/Cre, and Type II of KI/WT and KI/Cre mice. **b** qRT-PCR-measured levels of murine *Casp6* mRNA from WT/WT, WT/Cre, Type I KI/WT and KI/Cre, and Type II KI/WT and KI/Cre hippocampus, cortex, cerebellum, and liver mRNA normalized to 18S RNA. Data shows mean and s.e.m. Each symbol represents data from one mouse. **c** Western blot of hippocampal, cortical, cerebellar and liver proteins detected with LS-B477 anti-human Casp6 antibody (top panel), and Cell Signaling 9762 anti-mouse and human Casp6 (bottom panel). **d** Quantification of Casp6 protein levels detected by 9762 in (**c**) normalized to Casp6 levels in WT/WT. Data represents mean ± s.e.m. Statistical evaluations were done with one-way ANOVA (*p* < 0.0001) followed by Dunnett’s post-hoc analysis vs WT/WT **p < 0.01, ***p < 0.001, **** < 0.0001 **e** Human Casp6 immunohistological staining with LS-B477 in hippocampal CA1 (SO; stratum oriens, PCL: pyramidal cell layer, SR: stratum radiatum, SLM: stratum lacunosum molecular), cortex, corpus callosum, fimbria and columns of the fornix regions from Type I and Type II KI/WT and KI/Cre mice. Bar = 50 μm. Arrows indicate immunopositive neurons and neurites. Arrow heads indicate dot-like staining. **f** Red safe-stained agarose gel of PCR amplified human *CASP6* transgene STOP sequence and CaMKIIα-Cre transgenes from WT/WT, WT/Cre, Type I KI/WT and KI/Cre (ACL) and Type II KI/WT and KI/Cre (ACL/G) hippocampus (H) and tail (T) DNA

Validation of the expression of *CASP6* was done after behavioral assessments upon sacrifice of the mice but we present it first because of unexpected findings as described below. Human *CASP6* mRNA transcript and protein levels were evaluated in the hippocampus, cortex and cerebellum of KI/Cre and control KI/WT, WT/Cre, or WT/WT littermate mice by RT-PCR and western blot analyses. *CASP6* mRNA was detected in the KI/Cre hippocampus, cortex and cerebellum but not in the WT/WT and WT/Cre negative controls, as expected (Fig. 1a). Surprisingly, while some KI/WT mice did not express human *CASP6* mRNA (labeled Type I) as expected, others did express *CASP6* mRNA (labelled Type II). Murine *Casp6* mRNA was expressed at similar levels in mice brains of all four genotypes, indicating that human transgene expression had no effect on the *Casp6* mouse mRNA levels (Fig. 1b). Consistently, human Casp6 protein was detected with the human-specific Casp6 antibody, LS-B477, in Type I KI/Cre but not KI/WT, and in type II KI/Cre and KI/WT (Fig. 1c top panel). Human Casp6 was not detected in Type I cerebellum but was detected in Type II cerebellum. Furthermore, human Casp6 was detected in the liver of Type II KI/WT and KI/Cre mice suggesting whole organ expression of the human transgene. Human and mouse Casp6 proteins could not be differentiated by size on western blots since the mouse Casp6 naturally lacks the pro-domain and the human *CASP6* transgene lacks the pro-domain to promote self-activation. Therefore, human and mouse Casp6 proteins were detected with the 9762 anti-mouse and human Casp6 antibody (Fig. 1c bottom panel). The western blots show increased expression of Casp6 protein in type I KI/Cre hippocampus and cortex and in type II KI/WT and KI/Cre hippocampus, cortex and cerebellum compared to negative controls. The results are consistent with high expression of hCasp6 compared to the endogenous mouse Casp6 expression (Fig. 1d). Immunohistochemical analyses of human Casp6 with the LS-B477 confirmed these expression patterns in situ (Fig. 1e & Additional file 1: Figure S1). That Casp6 expression was not limited to the hippocampus and cortex as expected from the T29–1 CaMKII-Cre recombinase mouse [50] and as we previously observed [29], was unsettling.

Further research of the literature revealed that the CAMKIIα gene is expressed in testis and expression of CAMKIIα-Cre recombinase can delete the STOP sequence of a floxed transgene and allow male germ line transmission of the STOP-excised transgene and thus whole body expression of a transgene in the F2 progeny [10]. Analyses of the human Casp6 transgene floxed STOP and excised STOP in tail and hippocampal DNA revealed the excision of the STOP sequence in only the hippocampal Type I KI/Cre DNA (Fig. 1f). No excision of the STOP sequence was observed in Type I KI/WT hippocampal or tail DNA, as expected. However, the STOP sequence of the human transgene was excised in hippocampal and tail DNA from Type II KI/WT and KI/Cre, independent of the presence of Cre transgene. These results confirm that Cre expression in the testis led to excision of the transgene in male germ cells and the excised transgene was transmitted to progeny.

Therefore, we named the Type I KI/Cre and KI/WT mice ACL, to be consistent with the previously published acronym of this line, and the Type II KI mice were named ACL/G for germ line transmission. All subsequent data was analysed taking differences between ACL and ACL/G mice into consideration.

### MB rescues Casp6-induced cognitive impairment in aged mice

**Fig. 2:**
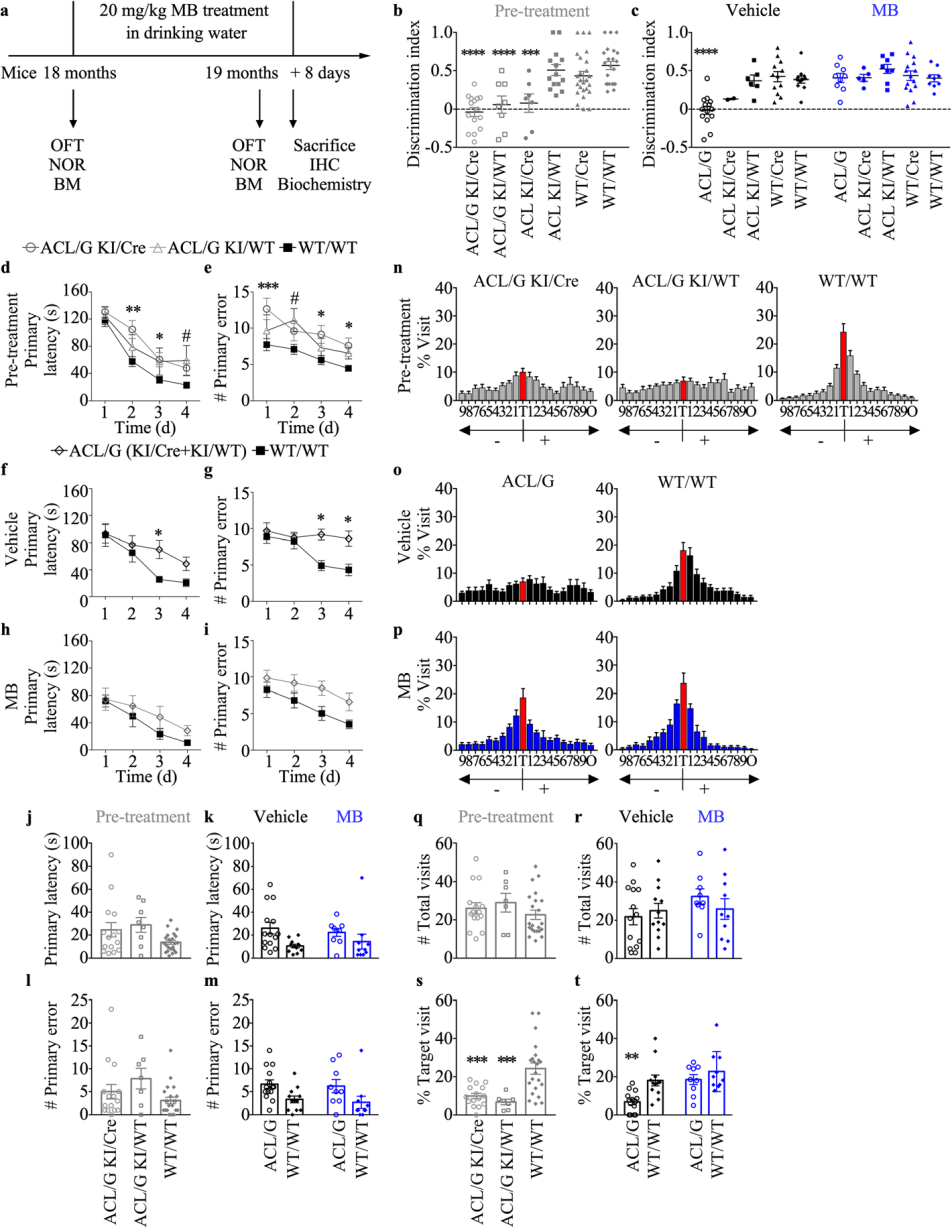
MB treatment reverses memory impairment in human Casp6-expressing mice. **a** Experimental design for drug administration and behavioral studies. OFT: open field test, NOR: novel object recognition, BM: Barnes maze. **b** and **c** NOR discrimination index of pre-treated ACL/G KI/Cre (*n* = 15) and KI/WT (*n* = 8), ACL KI/Cre (*n* = 7) and KI/WT (*n* = 14),WT/Cre (*n* = 25), WT/WT (*n* = 22) (**b**), vehicle-treated ACL/G KI/WT (*n* = 4) and KI/Cre (*n* = 10), ACL KI/Cre (*n* = 2), ACL KI/WT (*n* = 6), WT/Cre (*n* = 12), or WT/WT (*n* = 12) mice and MB-treated ACL/G KI/Cre (*n* = 5) and KI/WT (*n* = 4), ACL KI/Cre (*n* = 5), ACL KI/WT (*n* = 8), WT/Cre (*n* = 13), or WT/WT (*n* = 10) mice (**c**). Each mouse tested is represented by one symbol. Bars represent the mean and error bars the s.e.m. Statistical evaluations were conducted with one-way ANOVA and Dunnett’s post hoc analysis vs WT/WT (**b**) or vehicle-treated WT/WT (**c**) **p < 0.01, ***p < 0.001, ****p < 0.0001. **d**-**i** Primary latency (**d**,**e**,**h**) and primary errors (**f**,**g**,**i**) during spatial acquisition training in the Barnes maze task in pre-treated (**d**&**f**), vehicle-treated (**e**&**g**), and MB-treated (**h**&**i**) mice. Statistics were done with repeated-measures two-way ANOVA. Time *p* < 0.003 (**d**, **e**, **g**, **h**, **i**), genotype *p* = 0.03 (**d**, **e**, **f**, **g**, **i**). interaction *p* = 0.03 (**g**). Post-hoc Dunnett analyses:*p < 0.05, **p < 0.01, ***p < 0.001 ACL/G KI/Cre vs WT/WT, #*p* < 0.05 ACL/G KI/WT vs WT/WT. Probe test primary latency (**j**&**k**) and errors (**l**&**m**), % visit to each hole: T = target, holes − 1 to − 9 are to the left whereas + 1 to + 9 are to the right of T (**n**-**p**), number of total visits (**q**&**r**), % visit to the target hole (**s**&**t**). One symbol represents individual mous. Bars represent mean and error bars s.e.m. One-way ANOVA *p* = 0.0248 and *p* = 0.0005 (**m**, **t**), Brown-Forsythe ANOVA *p* = 0.0008 (**s**) with Dunnett’s post hoc analysis **p < 0.01, ***p < 0.001 vs WT/WT (**s**) or vehicle-treated WT/WT (**t**)

To evaluate if MB can reverse Casp6-induced cognitive impairment in aged animals, 18 month-old Casp6-expressing KI/Cre mice and negative control KI/WT, WT/Cre and WT/WT littermates were orally given approximately 20 mg/kg MB daily for 1 month. Mice were submitted to open field, NOR, and Barnes maze tasks before and after MB treatment to assess locomotion and anxiety, episodic memory, and spatial memory, respectively (Fig. 2a).

Casp6 expression in Type II ACL/G KI/Cre and KI/WT is the same (Fig. 1a&d) since the STOP sequence was excised in the germ line and the expressing Casp6 transgene is transmitted in a Cre-independent manner. Nevertheless, the behavioral data from pre-treated ACL/G KI/Cre and KI/WT are shown (Fig. 2b) to ensure that these two groups behave the same. Having confirmed that ACL/G KI/Cre and KI/WT behave the same, the ACL/G KI/Cre and KI/WT were grouped together and named ACL/G to distinguish them from the ACL. Before treatment, ACL/G KI/Cre and KI/WT, ACL KI/Cre and KI/WT, WT/Cre and WT/WT mice performed normally and equally well in the total distance traveled, % time moving, % used zones, moving speed, and % time in periphery of the open field apparatus indicating normal locomotor abilities and absence of anxiety amongst the different groups (Additional file 1: Figure S2). This indicates that genotype, handling, and treatment did not affect locomotion or anxiety in any mouse groups, thus enabling proper measurement of episodic and spatial memory in these mice.

MB treatment rescued Casp6-mediated episodic memory deficits measured by the novel object recognition (NOR) task. Compared to the ACL KI/WT, WT/Cre and WT/WT non Casp6-expressing control littermate groups, Casp6-expressing ACL/G KI/Cre and KI/WT and ACL KI/Cre mice exhibited significantly lower discrimination indices before treatment and after vehicle treatment (Fig. 2b&c). However, MB treatment increased discrimination indices to WT/WT levels in ACL/G and ACL KI/Cre mice whereas discrimination indices were not altered in the control littermate genotypes. Again, no significant difference in NOR was observed between ACL/G KI/Cre and KI/WT (Additional file 1: Figure S3a&b). MB had no effect on the locomotor or exploratory activities of these mice in the NOR apparatus, since the total numbers of touches to objects, distance travelled, % time moving, and % used zones were equivalent in each group of mice (Additional file 1: Figure S3c-f). These results indicate that 3 months after the onset of episodic memory deficits, a 1 month MB treatment reverses Casp6-induced episodic memory impairment.

Mice were submitted to the Barnes maze to assess spatial memory. Pre-treated and vehicle-treated ACL/G KI/Cre and KI/WT mice exhibited higher primary latency (Fig. 2d&e) and errors (Fig. 2f&g) in day 3 and 4 of acquisition training compared with the WT/WT genotype. This deficit was corrected with MB treatment in KI/Cre mice (Fig. 2h&i). No difference was observed between vehicle- or MB-treated ACL/G KI/Cre and ACL/G KI/WT in primary latency and primary error (Additional file 1: Figure S4a-d). During the Barnes maze probe, where the escape hatch has been blocked, there was no significant difference in primary latency (Fig. 2j&k) or primary errors (Fig. 2l&m) to find the target between pre-treated (Fig. 2j&l), vehicle- or MB-treated (Fig. 2k&m) ACL/G (KI/Cre and KI/WT) mice and WT/WT mice. However, the pre- (Fig. 2n) and vehicle-treated (Fig. 2o) ACL/G (KI/Cre and KI/WT) mice showed less discrimination of the target area than the WT/WT mice (Fig. 2n&o). MB reversed this deficit (Fig. 2p). The total # of visits to the holes of the Barnes apparatus did not change across these genotypes and treatments (Fig. 2q&r) but the % visit to the target hole was significantly lower in pre- and vehicle-treated ACL/G mice compared to WT/WT (Fig. 2s&t). Again, MB treatment normalized the % target visits in ACL/G mice. No difference was observed between vehicle- or MB-treated ACL/G KI/Cre and ACL/G KI/WT in primary latency (Additional file 1: Figure S4e&f), primary error (Additional file 1: Figure S4g&h), discrimination of the target area (Additional file 1: Figure S4i&j), # total visits (Additional file 1: Figure S4k&l), and % total visits (Additional file 1: Figure S4m&n).

To confirm the effect of MB on cognitive deficits was caused by the specific expression of hCasp6 in the hippocampus, ACL mice were assessed in the Barnes maze. No significant difference was observed in acquisition training across genotypes, treatment and days of acquisition training (Additional file 1: Figure S5a-f). During the Barnes maze probe, no significant difference was observed in primary latency or primary error across genotypes and treatments (Additional file 1: Figure S5g&h). This contrasts with the ACL/G mice, and suggests that the higher and diversified expression of Casp6 in ACL/G brains exacerbates cognitive deficits. However, the % visit to the target was severely decreased in the Casp6-expressing ACL KI/Cre mice compared to the other three control genotypes, and this deficit was reversed in MB-treated mice (Additional file 1: Figure S5i&j). The # total visits was similar in all genotypes and treatments (Additional file 1: Figure S5k). These results indicate that MB treatment for 1 month is effective in reversing Casp6-induced spatial memory impairment.

### MB reverses Casp6-induced synaptic dysfunction in aged mice

**Fig. 3:**
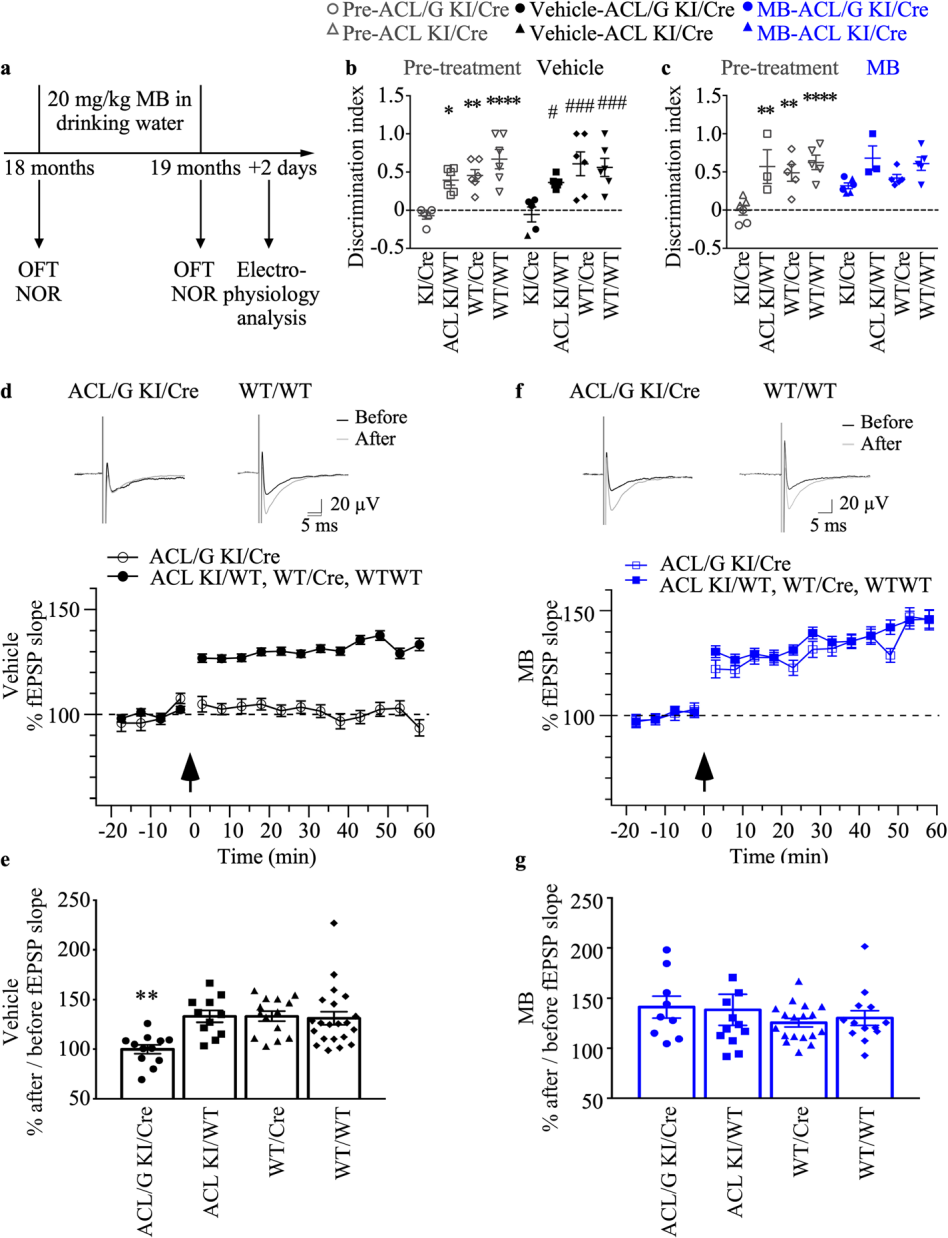
MB treatment improves synaptic function of the hippocampal Schaffer collateral-CA1 pathway in human Casp6 mice. **a** Experimental design for drug administration, behavioral and electrophysiological study. OFT: open field test, NOR: novel object recognition. **b** Discrimination NOR index in pre-treated and vehicle-treated KI/Cre (ACL/G *n* = 4, ACL *n* = 1), ACL KI/WT (*n* = 6), WT/Cre (*n* = 6), or WT/WT (*n* = 6) mice. Statistical evaluations were done by repeated-measures two-way ANOVA (genotype *p* < 0.0001, time *p* = 0.8324, interaction *p* = 0.4883) and post-hoc Tukey’s analyses pre-treated KI/Cre vs control groups in each treatment *p < 0.05, ** *p* < 0.01, *** *p* < 0.001, ****p < 0.0001, vehicle-treated KI/Cre vs control groups # *p* < 0.05, # # # *p* < 0.001, and pre-treated vs vehicle-treated (NS). **c** Discrimination NOR index in pre-treated and MB-treated KI/Cre (ACL/G *n* = 3, ACL *n* = 3), ACL KI/WT (*n* = 3), WT/Cre (*n* = 5), or WT/WT (*n* = 5) mice. Statistical evaluations as described in (b). Each symbol represents data from one individual mouse. **d**&**f** Top panel: representative traces before and after theta-burst stimulation in vehicle-treated (**d**) and MB-treated ACL/G KI/Cre (**f**) and WT/WT. **e** After/before ratio of the fEPSP slope in vehicle-treated mice (one-way ANOVA *p* = 0.0003; Dunnett’s post-hoc **p < 0.01 vs WT/WT). **d**&**f** Bottom panel: Extracellular fEPSP slope from acute slices of vehicle-treated ACL/G KI/Cre mice (99.967% ± 4.453%, *n* = 12 slices) and ACL KI/WT, WT/Cre or WT/WT (130.028% ± 3.6611%, *n* = 46 slices) (**d**) or MB-treated KI/Cre (141.226% ± 10.955%, *n* = 9 slices) and KI/WT, WT/Cre or WT/WT mice, control genotypes (132.424% ± 4.78%, *n* = 43 slices, *p* = 0.3936). **f** Arrows indicate time of theta-burst stimulation. The fEPSP recordings from vehicle-treated KI/WT (*n* = 11 slices), WT/Cre (*n* = 14 slices) or WT/WT (*n* = 21 slices) or MB-treated from KI/WT (*n* = 12 slices), WT/Cre (*n* = 18 slices) and WT/WT (*n* = 13 slices) were pooled since they were statistically indistinguishable. **g** After/before ratio of the fEPSP slope in in MB-treated mice (one-way ANOVA, *p* = 0.6223)

To evaluate whether MB reverses Casp6-mediated synaptic dysfunction in aged animals, we examined hippocampal synaptic plasticity by field recording in another group of mice exposed to vehicle or MB (Fig. 3a). All mice, submitted to electrophysiological analyses, were tested in open field and NOR tasks before and after vehicle or MB treatment. Because experimental animals were blinded, we only distinguished ACL/G from ACL mice after the experimentation. As a consequence, six Casp6-expressing mice (four ACL/G KI/Cre and two ACL KI/Cre) were treated with vehicle (Fig. 3b) and six Casp6-expressing mice (three ACL/G KI/Cre and three ACL KI/Cre) were treated with MB (Fig. 3c). The NOR discrimination indices in ACL/G or ACL KI/Cre mice were indistinguishable. These were compared to six ACL KI/WT, six WT/Cre, and six WT/WT vehicle-treated, and three ACL KI/WT, five WT/Cre, and five WT/WT MB-treated control mice. Furthermore, NOR behavior was compared the same mice before (pre-treatment) and after (vehicle or MB) treatment to evaluate changes due to treatment. As observed in Fig. 2b&c, pre- and vehicle-treated Casp6-expressing ACL/G and ACL KI/Cre mice displayed significantly lower discrimination indices, whereas MB-treated mice showed normal discrimination indices, compared with those of the ACL KI/WT, WT/Cre and WT/WT control littermate mice (Fig. 3b&c). There was no significant difference in the discrimination indices between the first (Fig. 2b&c) and second (Fig. 3b&c) set of experiments.

Synaptic plasticity at the Schaffer collateral-CA1 pathway was investigated in acute transverse hippocampal sections from Casp6-expressing ACL/G KI/Cre and control littermate mice. In vehicle-treated control mice, theta-burst induction resulted in ~ 30% LTP (130.028% ± 3.66%) that lasted at least 1 h (Fig. 3d). Casp6-expressing KI/Cre mice showed significantly less LTP (99.97% ± 4.45%) compared to littermate controls (Fig. 3d&e). This deficit was corrected in MB-treated KI/Cre mice (Fig. 3f&g). MB treatment thus rescued LTP in KI/Cre mice, indicating that MB may reverse Casp6-induced synaptic dysfunction at the hippocampal Schaffer collateral-CA1 pathway.

### MB inhibits Casp6 activity in the brain of aged mice

**Fig. 4:**
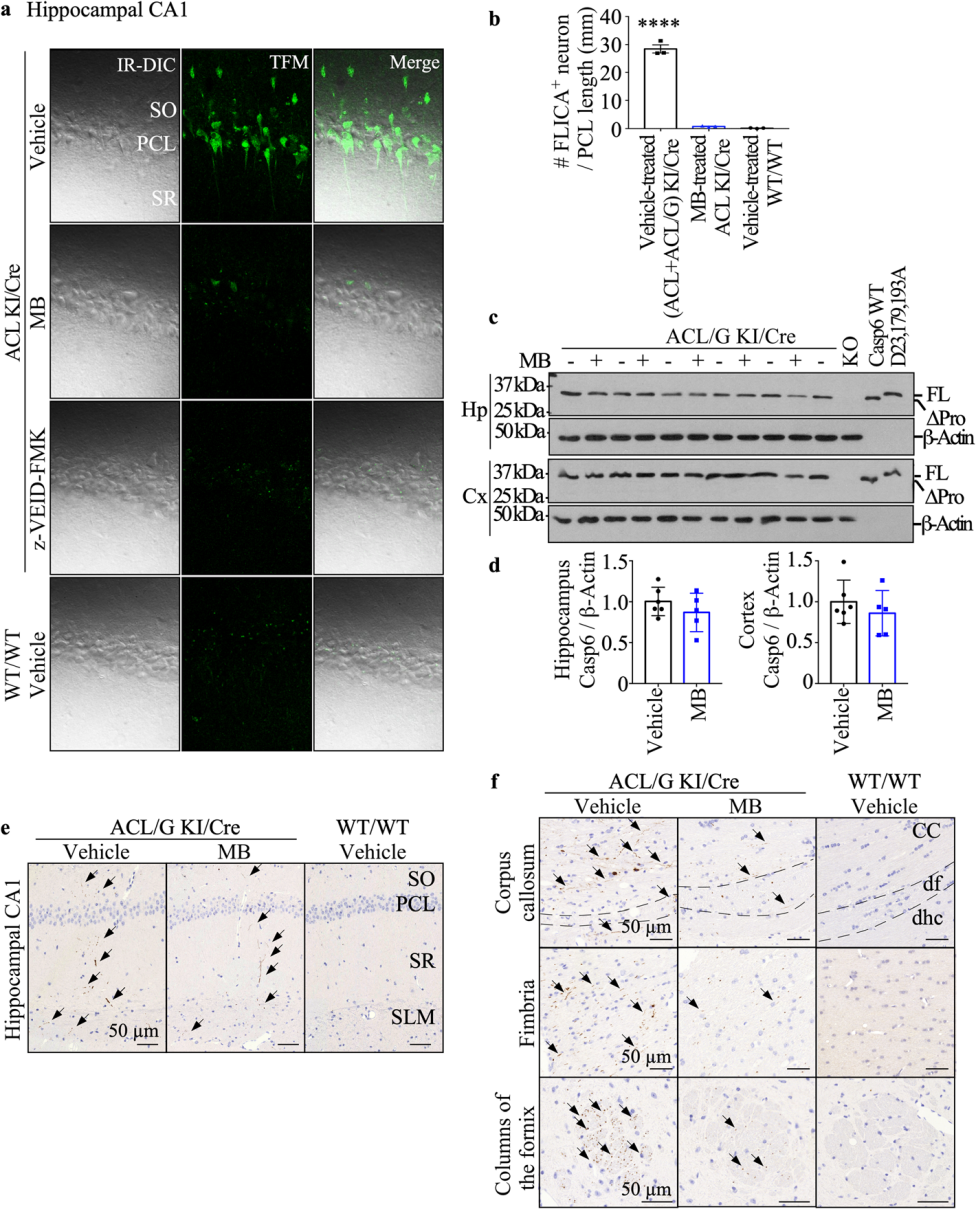
MB inhibits Casp6 activity in the brains of aged mice. **a** Representative two photon images of Casp6-FLICA stained CA1 neurons in acute brain slices from vehicle-treated KI/Cre (ACL/G *n* = 1, ACL *n* = 2), MB-treated KI/Cre (ACL *n* = 3), or WT/WT (*n* = 3) mice, and z-VEID-FMK-treated KI/Cre (ACL/G *n* = 1, ACL *n* = 2) mice. IR-DIC: infrared differential interference contrast, TFM: two-photon microscope. **b** Quantification of the numbers of FLICA positive neurons per mm along the pyramidal neuron. Data represents mean ± s.e.m. Statistical evaluations were done with one-way ANOVA (*p* = 0.0026) followed by Dunnett’s post-hoc analysis. ****p < 0.01 vs vehicle-treated WT/WT. **c** Western blotting and **d** quantification of Casp6 in hippocampus or cortex from vehicle- (*n* = 6) or MB-treated (*n* = 5) ACL/G KI/Cre mice. Uncleavable Casp6D23,179,193A recombinant protein, recombinant WT Casp6 protein, which has lost its pro-domain during purification, and Casp6 knock out mouse proteins were used as controls. Data shown as mean and s.e.m. Each symbol represents data from one mouse. **e**&**f** Representative micrographs of immunopositive Tub∆Casp6 in the SO, PCL, SR, and SLM CA1 regions (**e**), and in corpus callosum, fimbria, and fornix (**f**) of vehicle-treated ACL/G KI/Cre (*n* = 5) or WT/WT (*n* = 3) mice, and MB-treated ACL/G KI/Cre (*n* = 2) mice. Bar = 50 μm. dhc: hippocampal commissure, df: dorsal fornix; arrows indicate immunopositive neurites

To directly assess the effect of MB treatment on Casp6 activity in Casp6-expressing brains, Casp6-FLICA was used to fluorescently label active Casp6 in acute hippocampal slices from vehicle- and MB-treated ACL KI/Cre mice. Because slicing causes neuronal damage associated with caspase activation, measurements were done from the intact neurons at a − 30 μm depth from the slice surface (Additional file 1: Figure S6), the neuronal layer also chosen for whole cell recording in electrophysiological studies. Under two-photon microscopy at − 30 μm depth, large numbers of FLICA-labeled neurons were detected in ACL and ACL/G KI/Cre, but not in the WT/WT hippocampi (Fig. 4a&b). MB-treated ACL KI/Cre mice and Casp6 peptide inhibitor z-VEID-FMK-incubated KI/Cre hippocampi slices showed only a few FLICA-labeled neurons, indicating that MB effectively inhibited Casp6. Western blot analyses in ACL/G mice hippocampi and cortices showed that MB did not change Casp6 levels in the hippocampus and cortex (Fig. 4c&d), thereby excluding the possibility that cognitive improvement in MB-treated mice is caused by a reduction of the transgenic human *CASP6* expression.

To assess Casp6-mediated neurodegeneration, brain sections were immunostained with a neoepitope antiserum against Tubulin cleaved by Casp6 (Tub∆Casp6). The cleavage of Tubulin by Caspase-6 is identical to Tubulin cleaved by Granzyme B and the removal of the short acidic C-terminus of Tubulin causes a dramatic reorganization of the cytoskeleton in cells [3, 26]. In neurons, it has been shown that caspase cleavage of Tubulin is a common and sensitive marker of axonal degeneration [45]. Tub∆Casp6 immunopositive neurons are observed in vehicle-treated (*n* = 5 ACL/G and 1 ACL) KI/Cre stratum oriens (SO), pyramidal cell layer (PCL), stratum radiatum (SR), and stratum lacunosum molecular (SLM) of the hippocampal CA1 region (Fig. 4e). Only slightly less Tub∆Casp6 was observed in MB-treated KI/Cre (*n* = 2 ACL/G and 5 ACL). No Tub∆Casp6 immunopositivity was observed in untreated WT/WT (*n* = 3), WT/Cre (*n* = 3), or KI/WT (*n* = 3 ACL vehicle-treated; *n* = 3 ACL MB-treated) (Additional file 1: Figure S7). Quantification of the immunopositive staining could not be done, because the low immunopositivity did not meet the criteria for precise quantification [19]. Similarly, Tub∆Casp6 immunopositivity was also detected in the hippocampal fibre systems of the fimbria-fornix and hippocampal commissure, and in the corpus callosum of ACL/G Casp6-expressing mice but the levels were noticeably reduced in MB-treated ACL/G mice (Fig. 4f). These results indicate that MB reduces levels of Tub∆Casp6 indicative of Casp6 activity in Casp6-expressing brains. Together with the FLICA experiments, these results confirm inhibition of Casp6 by MB treatment in mice brains.

### Methylene blue decreases microglia numbers or subtypes in the hippocampal CA1 and in the hippocampal fibre system and white matter of Casp6-expressing mice

**Fig. 5:**
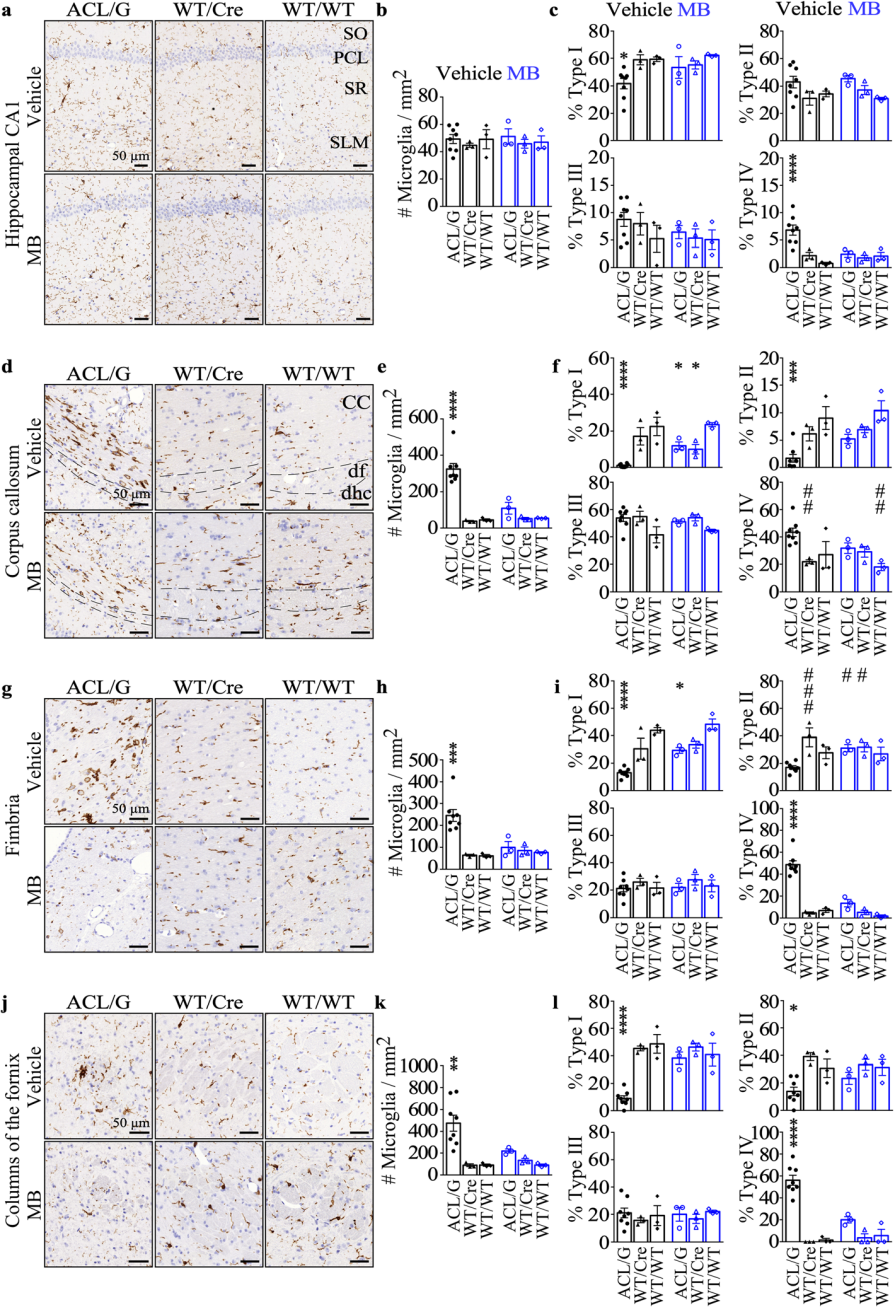
MB decreases microglial activation in aged Casp6 mice brains. Representative micrographs, Bar = 50 μm (**a**, **d**, **g**, **j**), quantitation of numbers of Iba1-immunopositive microglia numbers/mm^2^ (**b**, **e**, **h**, **k**) and subtype I-IV microglia (**c**, **f**, **i**, **l**) in hippocampus CA1 (**a**-**c**), corpus callosum (**d**-**f**), fimbria (**g**-**i**), and fornix (**j**-**l**) of vehicle-treated ACL/G (KI/Cre n = 5, KI/WT *n* = 3), WT/Cre (*n* = 3), WT/WT (*n* = 3) or MB-treated ACL/G (KI/Cre *n* = 2, KI/WT *n* = 1), WT/Cre (*n* = 3), and WT/WT (*n* = 3) mice. Data represents mean and s.e.m. Each symbol represents data from one mouse. Statistical analyses were conducted with one-way ANOVA or Brown-Forsythe ANOVA when variances were unequal, for e,h,k *p* < 0.0002, for Type I in (**c**,**i**,**l**) *p* < 0.02, for Type II in f,I,l *p* < 0.004, for Type IV in c,f,i *p* < 0.0.005; all others are NS. Post-hoc analyses were done with Dunnett’s test. *p < 0.05, **p < 0.01, ***p < 0.001, ****p < 0.0001 vs vehicle-treated WT/WT or vehicle-treated ACL/G # *p* < 0.05, # # *p* < 0.01, # # # *p* < 0.001

Immunohistochemical staining and quantitative analyses showed that the total number of Iba1 positive microglia in the entire CA1 region was similar in ACL/G and control genotypes, whether treated or not with MB (Fig. 5a&b). Quantitative morphological assessment of type I (ramified), type II (reactive), type III (amoeboid) and type IV (phagocytic) microglia (Additional file 1: Figure S8a) in the CA1 region indicated decreased type I, representative of resting microglia, and increased type IV, representative of phagocytic microglia, in ACL/G CA1 compared to WT/WT (Fig. 5c). MB restored normal levels of Type I and IV microglia in ACL/G CA1.

In the ACL/G mice corpus callosum, microglia numbers were increased seven-fold, and type I and II microglia decreased considerably. MB treatment normalized microglia and subtypes numbers (Fig. 5d-f). In the fimbria region (Fig. 5g-i) and in the fornix region (Fig. 5j-l), increased numbers of Iba1 immunopositive microglia (Fig. 5g&h,j&k), decreased type I or II and increased type IV microglia were observed (Fig. 5i&l). Again, these were normalized in MB treated mice. These results demonstrate that MB inhibited Casp6-induced microglial inflammation in the fibre systems of ACL/G mice. Unfortunately, only one vehicle-treated ACL KI/Cre brain was available for these analyses and therefore could not be quantified. However, five MB-treated ACL were assessed and comparison of ACL with ACL/G and control genotypes (Additional file 1: Figure S8b-m), indicated that ACL, unlike ACL/G, have inflammation limited to the hippocampal CA1 region since there is no difference between ACL KI/Cre and ACL KI/WT microglia numbers and subtypes in the corpus callosum, fimbria or fornix. Increased activated microglia subtypes III and IV in CA1 were normalized with MB treatment, and reached levels observed in MB-treated ACL/G.

Together, these results indicate that MB normalizes microglial numbers or subtypes when these are increased in hCasp6-expressing regions of the brain.

### Increased GFAP-immunopositive astrocytes in the hippocampal fibre system and white matter are normalized by MB in the ACL/G mice

**Fig. 6:**
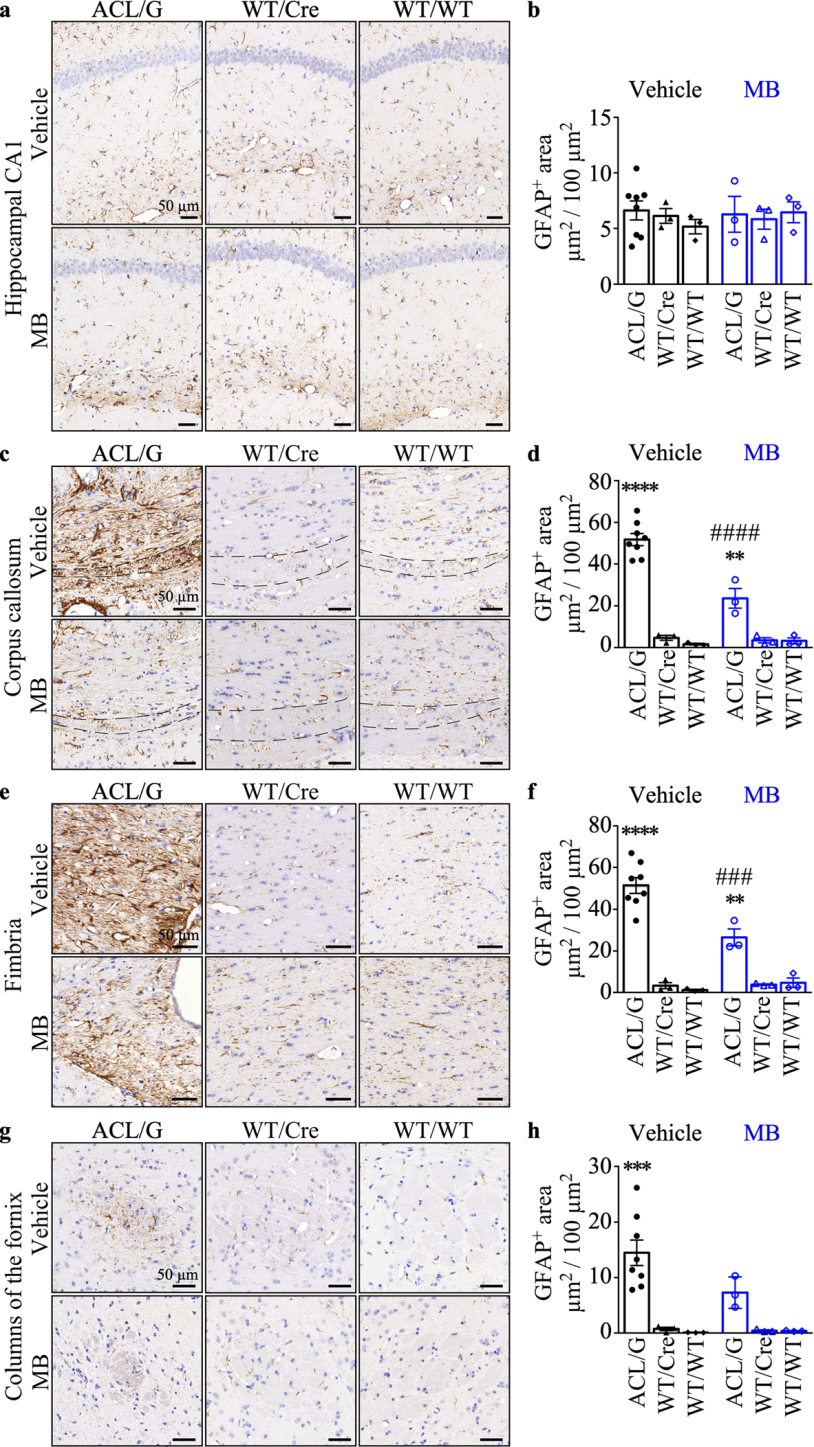
MB decreases activated astrocytes in the hippocampal fiber systems and the white matter of ACL/G Casp6 mice. Representative micrographs, bar = 50 μm (**a**, **c**, **e**, **g**) and quantitation (**b**, **d**, **f**, **h**) of GFAP-immunopositive astrocytes (μm^2^ immunopositive GFAP/100μm^2^ area) in hippocampus CA1 (**a**&**b**), corpus callosum (**c**&**d**), fimbria (**e**&**f**), and fornix (**g**&**h**) of vehicle-treated ACL/G (KI/Cre *n* = 5, KI/WT *n* = 3), WT/Cre (*n* = 3), WT/WT (*n* = 3) or MB-treated ACL/G (KI/Cre *n* = 2, KI/WT *n* = 1),WT/Cre (*n* = 3), and WT/WT (*n* = 3) mice. Data represents mean and s.e.m. Statistical evaluations were conducted with Brown-Forsythe ANOVA *p* < 0.001 (**d**, **f**, **h**) Post-hoc analyses were done with Dunnett’s test. ***p < 0.001, ****p < 0.0001 vs vehicle-treated WT/WT. # # #*p* < 0.001, # # # #*p* < 0.0001 vs vehicle-treated ACL/G

Immunohistochemical analyses showed no difference in astroglial glial fibrillary acidic protein (GFAP)-immunopositive levels in the hippocampal CA1 region of ACL/G and control genotypes, without or with MB treatment (Fig. 6a&b). Western blot analysis confirmed that GFAP levels were equivalent in the hippocampus (Additional file 1: Figure S9a&b). GFAP immunopositive astrocytes increased in the corpus callosum (Fig. 6c&d), fimbria (Fig. 6e&f), and fornix (Fig. 6g&h) of ACL/G mice and these were significantly reduced or normalized with MB. GFAP immunopositivity was unchanged in ACL KI/Cre CA1 region compared to ACL/G and ACL KI/WT negative control (Additional file 1: Figure S9c&d). As expected since *CASP6* expression is limited to the hippocampus in ACL KI/Cre, the levels of GFAP immunoreactivity were not significantly different from those in ACL KI/WT in the corpus callosum, fimbria and fornix (Additional file 1: Figure S9e-j). These results indicate increased astrocyte reactivity in Casp6-expressing hippocampal fibre system and white matter is normalized by MB treatment.

## Discussion

Here, the reversibility of Casp6-mediated brain damage by MB was investigated in the ACL transgenic mice expressing a knocked-in human *CASP*6 cDNA in the hippocampal CA1 region under CAMKIIα promoter-directed Cre recombinase [29], and in the ACL/G transgenic mice expressing human *CASP6* in all tissues due to the action of Cre recombinase in germ cells. The mice were treated at 18 months of age, 3 months after the onset of cognitive impairment [29]. The ability of MB, a caspase inhibitor [36] to inhibit Casp6 and reverse Casp6-mediated cognitive impairment, neuronal function, and inflammation was assessed after 1 month of treatment.

In vivo and in situ analyses indicated that MB can reverse cognitive impairment, neuroinflammation and neuronal dysfunction. We observed reversal of episodic memory and spatial memory impairment in Casp6-expressing mice after only 1 month of treatment. Reversal of cognitive impairment occurred in the ACL mouse model, where hCasp6 expression is mostly expressed in CA1 neurons [29, 50], and in the ACL/G mouse model, where hCasp6 is expressed everywhere. Immunohistochemical analyses of hCasp6-expressing brain areas indicated indirect evidence for the inhibition of Casp6 via decreased immunostaining of Tub∆Casp6 [26]. Unfortunately, Casp6 active subunits are very difficult to detect by immunohistochemistry in mice because of cross-reacting epitopes and by western blot because of the high turnover of the subunits which are degraded by the proteasome [49]. Nevertheless, microglial and astroglial activation are almost always associated with neuronal degeneration, which Casp6 activity can induce [12, 32, 44]. Here, MB normalized or significantly blunted Casp6-expression-mediated increases in Iba1-immunopositive microglial numbers and microglial subtypes, and GFAP-immunopositive astrocyte numbers. The MB reversal of neuroinflammation was particularly evident in the ACL/G mice brains, where hCasp6 expression was more widespread and neuroinflammation more severe. The idea of an important role of neuroinflammation in cognitive deficits is consistent with recent studies showing that inflammatory Casp1 inhibitors, elimination of *Casp1* gene expression by knock down or siRNAs, or elimination of Casp1-activating inflammasome pathways, reverse inflammation and cognitive deficits in AD mice models [15, 22, 47, 51]. This hypothesis is also supported by the association of peripheral inflammatory markers with brain atrophy and decreased cognition in aged individuals [53]. These results suggest that the effect of MB was related to inhibition of Casp6.

To confirm a direct effect of MB on neuronal function, electrophysiology was performed in hippocampal brain slices. MB normalized LTP induction in the hippocampal Schaffer collateral-CA1 pathway in acute brain slices of Casp6-expressing mice. In these hippocampal slices, we observed inhibition of Casp6 activity in MB-treated mice, with FLICA, thus confirming inhibition of Casp6 activity in MB-treated mice brains. An indirect effect on Casp6 expression levels was excluded, therefore confirming inhibition of Casp6 activity by MB in association with reversal of neuronal dysfunction.

The reversibility of hippocampal neuronal dysfunction in situ and cognitive impairment in vivo imply that hCasp6 does not cause neuronal cell death since this would be irreversible. This is consistent with our previous observations in human Alzheimer brains, where active Casp6 and Tau∆Casp6 were abundant in neurons that otherwise exhibited normal nuclear morphology and not the expected condensed chromatin of apoptotic neurons [20]. Furthermore, we and others have shown that high expression of active Casp6 in cells does not cause apoptosis or cell death [18, 25]. Nevertheless, Casp6 activity has been associated with axonal degeneration [12, 32, 43, 44]. Casp6 cleaves several human neuronal proteins that are essential to normal neuronal function [6, 20, 21, 26, 28], supporting its role in axonal degeneration. Given that Casp6 activity is highly abundant in human Alzheimer brains, that Casp6 levels in the human entorhinal cortex and hippocampal CA1 regions correlate with lower episodic and semantic memory performance in aged individuals, and that Casp6 activity leads to increased amyloid beta peptide from neurons, inhibition of Casp6, if done early enough, might be an efficient treatment against AD cognitive deficits and dementia.

We cannot exclude other mechanisms of action for MB in our mouse models. MB has pleiotropic effects. MB-mediated improvement of mitochondrial respiration and oxygen consumption has been suggested to be responsible for increased memory function in WT rodents [9, 41], although others did not detect an effect of chronic MB oral treatment on mitochondrial function in either WT or trigenic AD mice [30]. MB is also well-known to inhibit Tau aggregation [55], but Casp6-expressing mice do not show accumulated Tau aggregates [29]. Interestingly, spatial memory impairment measured by the Morris water maze in the Tau transgenic mouse was prevented, but not reversed, by MB [23, 34, 46]. Therefore, given our mouse models, it is likely that the positive action of MB is due to the inhibition of Casp6 activity.

Lastly, this is the first report of a Casp6 expression effect on neuritic degeneration in the white matter, consistent with the atrophy observed in MCI, and mild to severe AD individuals by magnetic resonance imaging or post-mortem pathological analyses [42, 52]. It is reported that white matter atrophy precedes neocortical atrophy and correlates with lower cognitive scores [56]. In addition, individuals who do not show cognitive impairment, but have the classical Aβ plaques and NTFs AD pathologies, only show atrophy in the white matter [14]. Therefore, neuritic degeneration in the white matter may be involved in AD pathophysiology. The re-establishment of the hippocampal intrinsic network by MB could thus reverse Casp6-mediated cognitive impairment.

## Conclusion

The effects of MB on Casp6-mediated neurodegeneration and neuroinflammation can be added to the pleiotropic actions of MB that would be beneficial against AD. Most importantly, MB has the ability to reverse Casp6-mediated cognitive deficits, neurodegeneration and neuroinflammation.

## Supplementary information

**Additional file 1: Figure S1.** Human Casp6 immunostaining by LS-B477 antibody. **Figure S2.** MB does not affect locomotor ability or anxiety assessed by open field test. **Figure S3.** NOR discrimination index and open-field locomotor ability between ACL/G or ACL KI/Cre and KI/WT. **Figure S4.** No difference was seen between ACL/G KI/Cre and ACL/G KI/WT during Barnes maze. **Figure S5.** MB reversed spatial memory impairments of ACL KI/Cre mice during Barnes maze. **Figure S6.** FLICA-Casp6 activity in acute brain slice CA1 neurons. **Figure S7.** Tub∆Casp6 immunostaining in hippocampal CA1 and fiber tracts. **Figure S8.** Microglial activation in hippocampal CA1 region and fiber tracts. **Figure S9.** Astroglial activation in Casp6-expressing brains.

## Data Availability

The datasets used and/or analysed during the current study are available from the corresponding author upon reasonable request. All data, our mice Casp6 model, and laboratory-generated materials such as antibodies will be made available upon request. Behavioral data recorded by the HVS system, histological slide scans on the Mirax scanner, electrophysiological data, are stored on external hard drives and laboratory computers and can be transferred upon request if requester can provide either a website for transfer or a hard drive with sufficient memory for the transfers.

## Abbreviations

ACSF: Artificial cerebrospinal fluid
AD: Alzheimer disease
ANOVA: Analysis of variance
Aβ: Amyloid-beta peptide
CAG: CMV immediate early enhancer/chicken β-Actin promoter fusion
CAMKIIa: Calmodulin kinase IIa
Casp6: Caspase-6
DMSO: Dimethyl sulfoxide
EDTA: Ethylenediaminetetraacetic acid
EPSP: Excitatory postsynaptic potential
FAM-VEID-fmk: Carboxyfluorescein-Val-Glu-Ile-Asp-fluoromethyl ketones
FLICA: Fluorescent labelled inhibitor of caspase
GFAP: Glial fibrillary acidic protein
Iba1: Ionized calcium binding adapter molecule 1
IC50: Half maximal inhibitory concentrations
KI: Knock in
LTP: Long term potentiation
MB: Methylene blue
MCI: Mild cognitively impaired
NCI: Non cognitively impaired
NFT: Neurofibrillary tangles
NMDG: N-Methyl-D-glucamine
NOR: Novel object recognition
PBS: Phosphate-buffered saline
PEG: Polyethylene glycol
PIPES: Piperazine-N, N-bis (2-ethanesulfonic acid)
TubΔCasp6: Tubulin cleaved by Casp6
WT: Wild type
Z-VEID-fmk: N-benzyloxycarbonyl-Val-Glu-Ile-Asp-(O-methyl)-fluoromethylketone

## References

[1] Abrahamsson T, Chou CYC, Li SY, Mancino A, Costa RP, Brock JA (2017). Differential regulation of evoked and spontaneous release by presynaptic NMDA receptors. Neuron.

[2] Abrahamsson Therese, Lalanne Txomin, Watt Alanna J., Sjöström P. Jesper (2016). Long-Term Potentiation by Theta-Burst Stimulation Using Extracellular Field Potential Recordings in Acute Hippocampal Slices. Cold Spring Harbor Protocols.

[3] Adrain C, Duriez PJ, Brumatti G, Delivani P, Martin SJ (2006). The cytotoxic lymphocyte protease, granzyme B, targets the cytoskeleton and perturbs microtubule polymerization dynamics. J Biol Chem.

[4] Ahmed Z, Sheng H, Xu YF, Lin WL, Innes AE, Gass J (2010). Accelerated lipofuscinosis and ubiquitination in granulin knockout mice suggest a role for progranulin in successful aging. Am J Pathol.

[5] Albrecht S, Bogdanovic N, Ghetti B, Winblad B, LeBlanc AC (2009). Caspase-6 activation in familial Alzheimer disease brains carrying amyloid precursor protein or presenilin I or presenilin II mutations. J Neuropathol Exp Neurol.

[6] Albrecht S, Bourdeau M, Bennett D, Mufson EJ, Bhattacharjee M, LeBlanc AC (2007). Activation of caspase-6 in aging and mild cognitive impairment. Am J Pathol.

[7] Aljanabi SM, Martinez I (1997). Universal and rapid salt-extraction of high quality genomic DNA for PCR-based techniques. Nucleic Acids Res.

[8] Braak H, Braak E (1991). Neuropathological stageing of Alzheimer-related changes. Acta Neuropathol.

[9] Callaway NL, Riha PD, Bruchey AK, Munshi Z, Gonzalez-Lima F (2004). Methylene blue improves brain oxidative metabolism and memory retention in rats. Pharmacol Biochem Behav.

[10] Choi CI, Yoon SP, Choi JM, Kim SS, Lee YD, Birnbaumer L (2014). Simultaneous deletion of floxed genes mediated by CaMKIIalpha-Cre in the brain and in male germ cells: application to conditional and conventional disruption of Goalpha. Exp Mol Med.

[11] Clifton J, Leikin JB (2003). Methylene blue. Am J Ther.

[12] Cusack CL, Swahari V, Hampton Henley W, Michael Ramsey J, Deshmukh M (2013). Distinct pathways mediate axon degeneration during apoptosis and axon-specific pruning. Nat Commun.

[13] de Calignon A, Fox LM, Pitstick R, Carlson GA, Bacskai BJ, Spires-Jones TL (2010). Caspase activation precedes and leads to tangles. Nature.

[14] de la Monte SM (1989). Quantitation of cerebral atrophy in preclinical and end-stage Alzheimer’s disease. Ann Neurol.

[15] Flores J, Noel A, Foveau B, Lynham J, Lecrux C, LeBlanc A (2018) Caspase-1 inhibition alleviates cognitive impairment, inflammation and amyloid accumulation in an Alzheimer’s disease mouse model. Nat Commun In press 10.1038/s41467-018-06449-x PMC6156230 30254377

[16] Gamblin TC, Chen F, Zambrano A, Abraha A, Lagalwar S, Guillozet AL (2003). Caspase cleavage of tau: linking amyloid and neurofibrillary tangles in Alzheimer’s disease. Proc Natl Acad Sci U S A.

[17] Gauthier S, Feldman HH, Schneider LS, Wilcock GK, Frisoni GB, Hardlund JH (2016). Efficacy and safety of tau-aggregation inhibitor therapy in patients with mild or moderate Alzheimer’s disease: a randomised, controlled, double-blind, parallel-arm, phase 3 trial. Lancet.

[18] Gray DC, Mahrus S, Wells JA (2010). Activation of specific apoptotic caspases with an engineered small-molecule-activated protease. Cell.

[19] Gundersen HJ, Jensen EB (1987). The efficiency of systematic sampling in stereology and its prediction. J Microsc.

[20] Guo H, Albrecht S, Bourdeau M, Petzke T, Bergeron C, LeBlanc AC (2004). Active caspase-6 and caspase-6-cleaved tau in neuropil threads, neuritic plaques, and neurofibrillary tangles of Alzheimer’s disease. Am J Pathol.

[21] Halawani D, Tessier S, Anzellotti D, Bennett DA, Latterich M, LeBlanc AC (2010). Identification of Caspase-6-mediated processing of the valosin containing protein (p97) in Alzheimer’s disease: a novel link to dysfunction in ubiquitin proteasome system-mediated protein degradation. J Neurosci.

[22] Heneka MT, Kummer MP, Stutz A, Delekate A, Schwartz S, Vieira-Saecker A (2013). NLRP3 is activated in Alzheimer’s disease and contributes to pathology in APP/PS1 mice. Nature.

[23] Hochgrafe K, Sydow A, Matenia D, Cadinu D, Konen S, Petrova O (2015). Preventive methylene blue treatment preserves cognition in mice expressing full-length pro-aggregant human tau. Acta Neuropathol Commun.

[24] Kaushal V, Dye R, Pakavathkumar P, Foveau B, Flores J, Hyman B (2015). Neuronal NLRP1 inflammasome activation of Caspase-1 coordinately regulates inflammatory interleukin-1-beta production and axonal degeneration-associated Caspase-6 activation. Cell Death Differ.

[25] Klaiman G, Champagne N, LeBlanc AC (2009). Self-activation of Caspase-6 in vitro and in vivo: Caspase-6 activation does not induce cell death in HEK293T cells. Biochim Biophys Acta.

[26] Klaiman G, Petzke TL, Hammond J, LeBlanc AC (2008). Targets of caspase-6 activity in human neurons and Alzheimer disease. Mol Cell Proteomics.

[27] LeBlanc A (1995). Increased production of 4 kDa amyloid beta peptide in serum deprived human primary neuron cultures: possible involvement of apoptosis. J Neurosci.

[28] LeBlanc A, Liu H, Goodyer C, Bergeron C, Hammond J (1999). Caspase-6 role in apoptosis of human neurons, amyloidogenesis, and Alzheimer’s disease. J Biol Chem.

[29] LeBlanc AC, Ramcharitar J, Afonso V, Hamel E, Bennett DA, Pakavathkumar P (2014). Caspase-6 activity in the CA1 region of the hippocampus induces age-dependent memory impairment. Cell Death Differ.

[30] Medina DX, Caccamo A, Oddo S (2011). Methylene blue reduces abeta levels and rescues early cognitive deficit by increasing proteasome activity. Brain Pathol.

[31] National Toxicology P (2008) Toxicology and carcinogenesis studies of methylene blue trihydrate (Cas No. 7220-79-3) in F344/N rats and B6C3F1 mice (gavage studies). Natl Toxicol Program Tech Rep Ser 540:1–224 18685714

[32] Nikolaev A, McLaughlin T, O'Leary DD, Tessier-Lavigne M (2009). APP binds DR6 to trigger axon pruning and neuron death via distinct caspases. Nature.

[33] Noël Anastasia, Zhou Libin, Foveau Bénédicte, Sjöström P. Jesper, LeBlanc Andréa C. (2018). Differential susceptibility of striatal, hippocampal and cortical neurons to Caspase-6. Cell Death & Differentiation.

[34] O'Leary JC, Li Q, Marinec P, Blair LJ, Congdon EE, Johnson AG (2010). Phenothiazine-mediated rescue of cognition in tau transgenic mice requires neuroprotection and reduced soluble tau burden. Mol Neurodegener.

[35] Pakavathkumar P, Noel A, Lecrux C, Tubeleviciute-Aydin A, Hamel E, Ahlfors JE (2017). Caspase vinyl sulfone small molecule inhibitors prevent axonal degeneration in human neurons and reverse cognitive impairment in Caspase-6-overexpressing mice. Mol Neurodegener.

[36] Pakavathkumar P, Sharma G, Kaushal V, Foveau B, LeBlanc AC (2015). Methylene blue inhibits Caspases by oxidation of the catalytic cysteine. Sci Rep.

[37] Peter C, Hongwan D, Kupfer A, Lauterburg BH (2000). Pharmacokinetics and organ distribution of intravenous and oral methylene blue. Eur J Clin Pharmacol.

[38] Pfaffl MW (2001). A new mathematical model for relative quantification in real-time RT-PCR. Nucleic Acids Res.

[39] Ramcharitar J, Afonso VM, Albrecht S, Bennett DA, LeBlanc AC (2013). Caspase-6 activity predicts lower episodic memory ability in aged individuals. Neurobiol Aging.

[40] Ramcharitar J, Albrecht S, Afonso VM, Kaushal V, Bennett DA, LeBlanc AC (2013). Cerebrospinal fluid tau cleaved by caspase-6 reflects brain levels and cognition in aging and Alzheimer disease. J Neuropathol Exp Neurol.

[41] Riha PD, Bruchey AK, Echevarria DJ, Gonzalez-Lima F (2005). Memory facilitation by methylene blue: dose-dependent effect on behavior and brain oxygen consumption. Eur J Pharmacol.

[42] Salat DH, Greve DN, Pacheco JL, Quinn BT, Helmer KG, Buckner RL (2009). Regional white matter volume differences in nondemented aging and Alzheimer’s disease. Neuroimage.

[43] Simon DJ, Weimer RM, McLaughlin T, Kallop D, Stanger K, Yang J (2012). A caspase cascade regulating developmental axon degeneration. J Neurosci.

[44] Sivananthan SN, Lee AW, Goodyer CG, LeBlanc AC (2010). Familial amyloid precursor protein mutants cause caspase-6-dependent but amyloid beta-peptide-independent neuronal degeneration in primary human neuron cultures. Cell Death Dis.

[45] Sokolowski JD, Gamage KK, Heffron DS, LeBlanc AC, Deppmann CD, Mandell JW (2014). Caspase-mediated cleavage of actin and tubulin is a common feature and sensitive marker of axonal degeneration in neural development and injury. Acta Neuropathol Commun.

[46] Spires-Jones TL, Friedman T, Pitstick R, Polydoro M, Roe A, Carlson GA (2014). Methylene blue does not reverse existing neurofibrillary tangle pathology in the rTg4510 mouse model of tauopathy. Neurosci Lett.

[47] Tan MS, Tan L, Jiang T, Zhu XC, Wang HF, Jia CD (2014). Amyloid-beta induces NLRP1-dependent neuronal pyroptosis in models of Alzheimer’s disease. Cell Death Dis.

[48] Ting JT, Daigle TL, Chen Q, Feng G (2014). Acute brain slice methods for adult and aging animals: application of targeted patch clamp analysis and optogenetics. Methods Mol Biol.

[49] Tounekti O, Zhang Y, Klaiman G, Goodyer CG, LeBlanc A (2004). Proteasomal degradation of caspase-6 in 17beta-estradiol-treated neurons. J Neurochem.

[50] Tsien JZ, Chen DF, Gerber D, Tom C, Mercer EH, Anderson DJ (1996). Subregion- and cell type-restricted gene knockout in mouse brain. Cell.

[51] Venegas C, Kumar S, Franklin BS, Dierkes T, Brinkschulte R, Tejera D (2017). Microglia-derived ASC specks cross-seed amyloid-beta in Alzheimer’s disease. Nature.

[52] Villain N, Desgranges B, Viader F, de la Sayette V, Mezenge F, Landeau B (2008). Relationships between hippocampal atrophy, white matter disruption, and gray matter hypometabolism in Alzheimer’s disease. J Neurosci.

[53] Walker Keenan A., Hoogeveen Ron C., Folsom Aaron R., Ballantyne Christie M., Knopman David S., Windham B. Gwen, Jack Clifford R., Gottesman Rebecca F. (2017). Midlife systemic inflammatory markers are associated with late-life brain volume. Neurology.

[54] Wilcock GK, Gauthier S, Frisoni GB, Jia J, Hardlund JH, Moebius HJ (2018). Potential of low dose Leuco-Methylthioninium Bis(Hydromethanesulphonate) (LMTM) monotherapy for treatment of mild Alzheimer’s disease: cohort analysis as modified primary outcome in a phase III clinical trial. J Alzheimers Dis.

[55] Wischik CM, Edwards PC, Lai RY, Roth M, Harrington CR (1996). Selective inhibition of Alzheimer disease-like tau aggregation by phenothiazines. Proc Natl Acad Sci U S A.

[56] Zhang Y, Schuff N, Jahng GH, Bayne W, Mori S, Schad L (2007). Diffusion tensor imaging of cingulum fibers in mild cognitive impairment and Alzheimer disease. Neurology.

