# Supplementary material for: Methylene blue inhibits Caspase-6 activity, and reverses Caspase-6-induced cognitive impairment and neuroinflammation in aged mice
Source: Acta Neuropathol Commun. 2019 Dec 16;7:210. doi: 10.1186/s40478-019-0856-6 (PMC6915996; doi:10.1186/s40478-019-0856-6)
Supplement: Supplementary file 1 — Additional file 1: Figure S1. Human Casp6 immunostaining by LS-B477 antibody. Figure S2. MB does not affect locomotor ability or anxiety assessed by open field test. Figure S3. NOR discrimination index and open-field locomotor ability between ACL/G or ACL KI/Cre and KI/WT. Figure S4. No difference was seen between ACL/G KI/Cre and ACL/G KI/WT during Barnes maze. Figure S5. MB reversed spatial memory impairments of ACL KI/Cre mice during Barnes maze. Figure S6. FLICA-Casp6 activity in acute brain slice CA1 neurons. Figure S7. Tub∆Casp6 immunostaining in hippocampal CA1 and fiber tracts. Figure S8. Microglial activation in hippocampal CA1 region and fiber tracts. Figure S9. Astroglial activation in Casp6-expressing brains. [file 40478_2019_856_MOESM1_ESM.docx]

**Additional file 1**

***Methylene blue inhibits Caspase-6 activity, and reverses Caspase-6-induced cognitive impairment and neuroinflammation in aged mice***

Libin Zhou^1,2^, Joseph Flores^1^, Anastasia Noël^1^, Olivier Beauchet^1,3,4^, P. Jesper Sjöström^5^, and Andrea C. LeBlanc^1,2,6*^

^1^Lady Davis Institute for Medical Research at Jewish General Hospital, 3999 Ch. Côte Ste-Catherine, Montreal, QC H3T 1E2, Canada

^2^Department of Anatomy and Cell Biology, McGill University, 3640 University Street Strathcona Anatomy Building, Montreal, QC H3A 0C7, Canada

^3^Department of Medicine, Division of Geriatric Medicine, Sir Mortimer B. Davis - Jewish General Hospital, 3999 Ch. Côte Ste-Catherine, Montreal, QC H3T 1E2, Canada

^4^ Lee Kong Chian School of Medicine, Nanyang Technological University, Singapore

^5^Centre for Research in Neuroscience, the BRaIN Program, Department of Neurology and Neurosurgery, McGill University, The Research Institute of the McGill University Health Centre, Montreal General Hospital, 1650 Cedar Avenue Montreal, QC H3G 1A4, Canada

^6^Department of Neurology and Neurosurgery, McGill University, 845 Sherbrooke O, Montreal, QC H3A 0G4, Canada.

Libin Zhou: libin.zhou@mail.mcgill.ca

P. Jesper Sjöström: jesper.sjostrom@mcgill.ca

Andrea C. LeBlanc: [andrea.leblanc@mcgill.ca](mailto:andrea.leblanc@mcgill.ca)

*Corresponding author: Andrea LeBlanc, PhD, Bloomfield Center for Research in Aging, Lady Davis Institute for Medical Research, Sir Mortimer B Davis Jewish General Hospital, 3755 ch. Côte Ste-Catherine, Montréal, QC, Canada H3T 1E2. Tel.: +1 (514) 340 8222 ext 24976. e-mail address: [andrea.leblanc@mcgill.ca](mailto:andrea.leblanc@mcgill.ca)

**
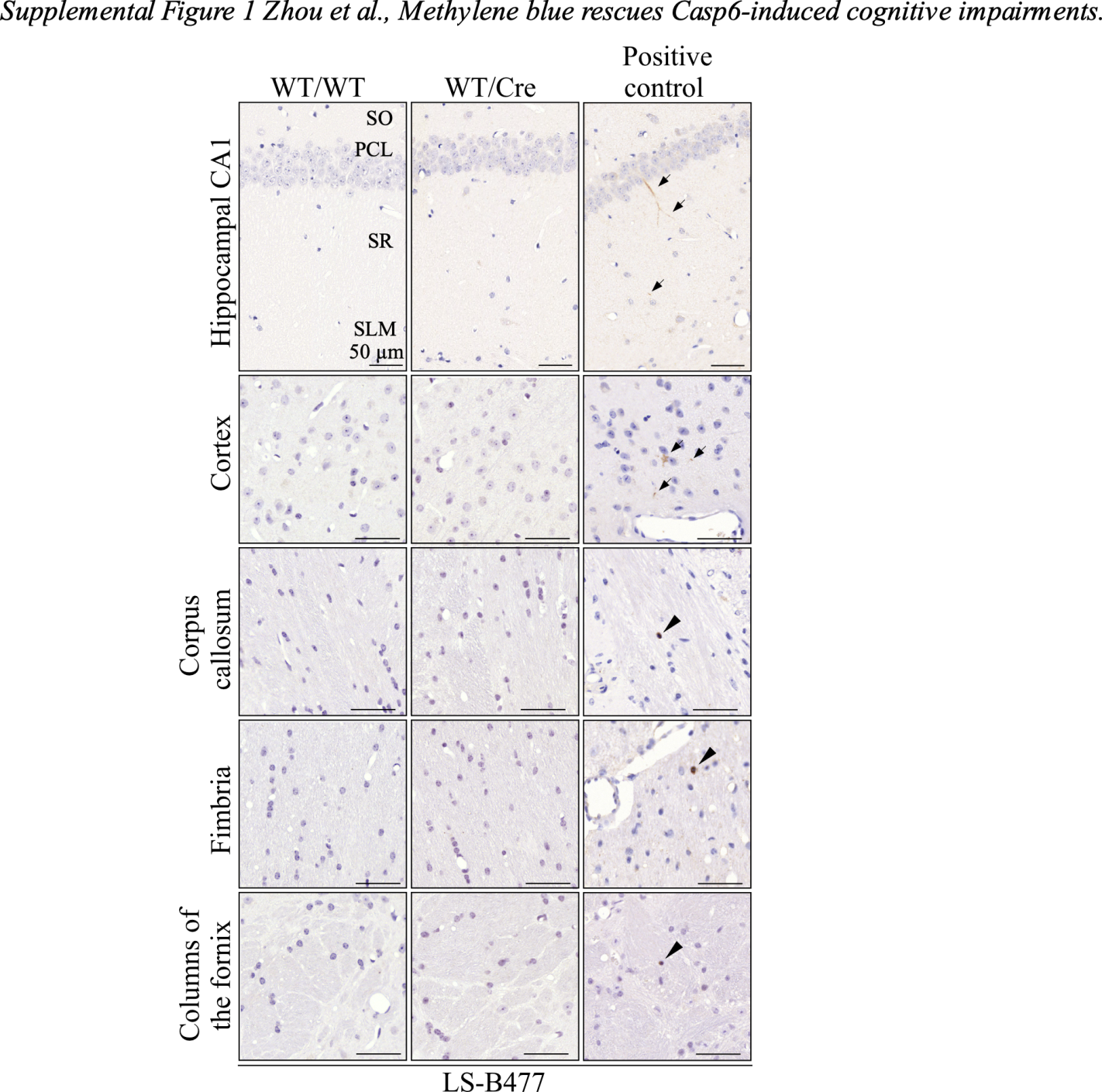
**

**Figure S1. Human Casp6 immunostaining by LS-B477 antibody.** Immunohistochemistry analysis shows no Casp6 distribution in the hippocampal CA1, cortex, the white matter of corpus callosum, hippocampal fimbria or fornix of WT/WT or WT/Cre mice. Positive control ACL/G KI/Cre shows the immunoreactivity in the neurites of CA1 (arrow) and the soma and neurites of cortex (arrow), and in the dot-like staining (arrowhead) of corpus callosum, fimbria, and the columns of the fornix. Bar = 50 µm.


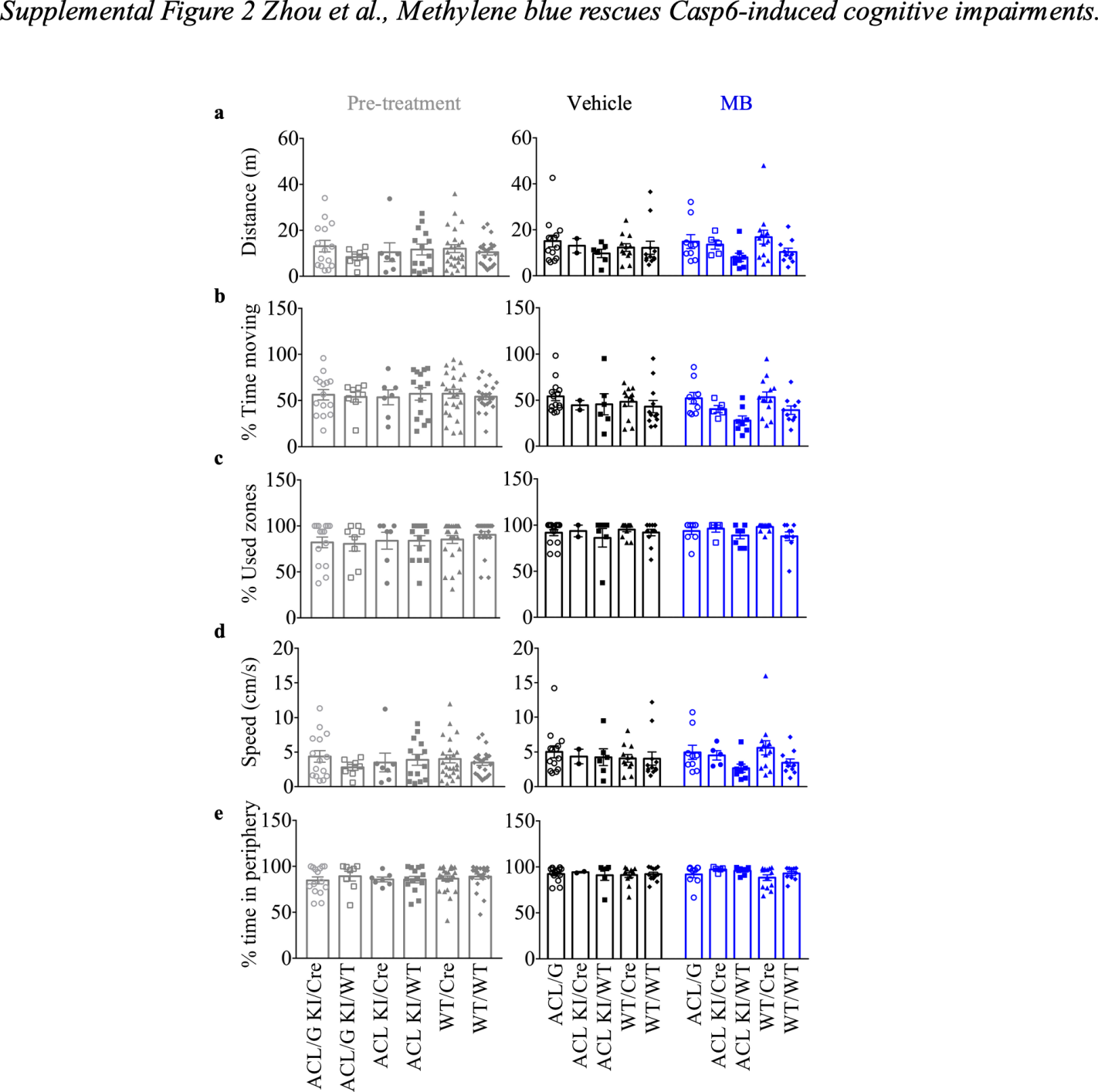


**Figure S2. MB does not affect locomotor ability or anxiety assessed by open field test.** The distance travelled (**a**), % time moving (**b**), % used zones (**c**), moving speed (**d**) and % time in periphery (**e**) of ACL/G KI/Cre (n=15), ACL/G KI/WT (n=8), ACL KI/Cre (n=7), ACL KI/WT (n=14), WT/Cre (n=25), or WT/WT (n=22) mice before treatment (pre-treatment), ACL/G (n=15), ACL KI/Cre (n=2), ACL KI/WT (n=6),WT/Cre (n=12), or WT/WT (n=12) mice after 1 month of treatment with vehicle, and ACL/G (n=9), ACL KI/Cre (n=5), ACL KI/WT (n=8), WT/Cre (n=13), or WT/WT (n=10) mice after 1 month of MB treatment, measured during the open field test. Each mouse tested is represented by one symbol. Bars represent the mean and error bars the s.e.m. No statistical difference was shown in one-way ANOVA.


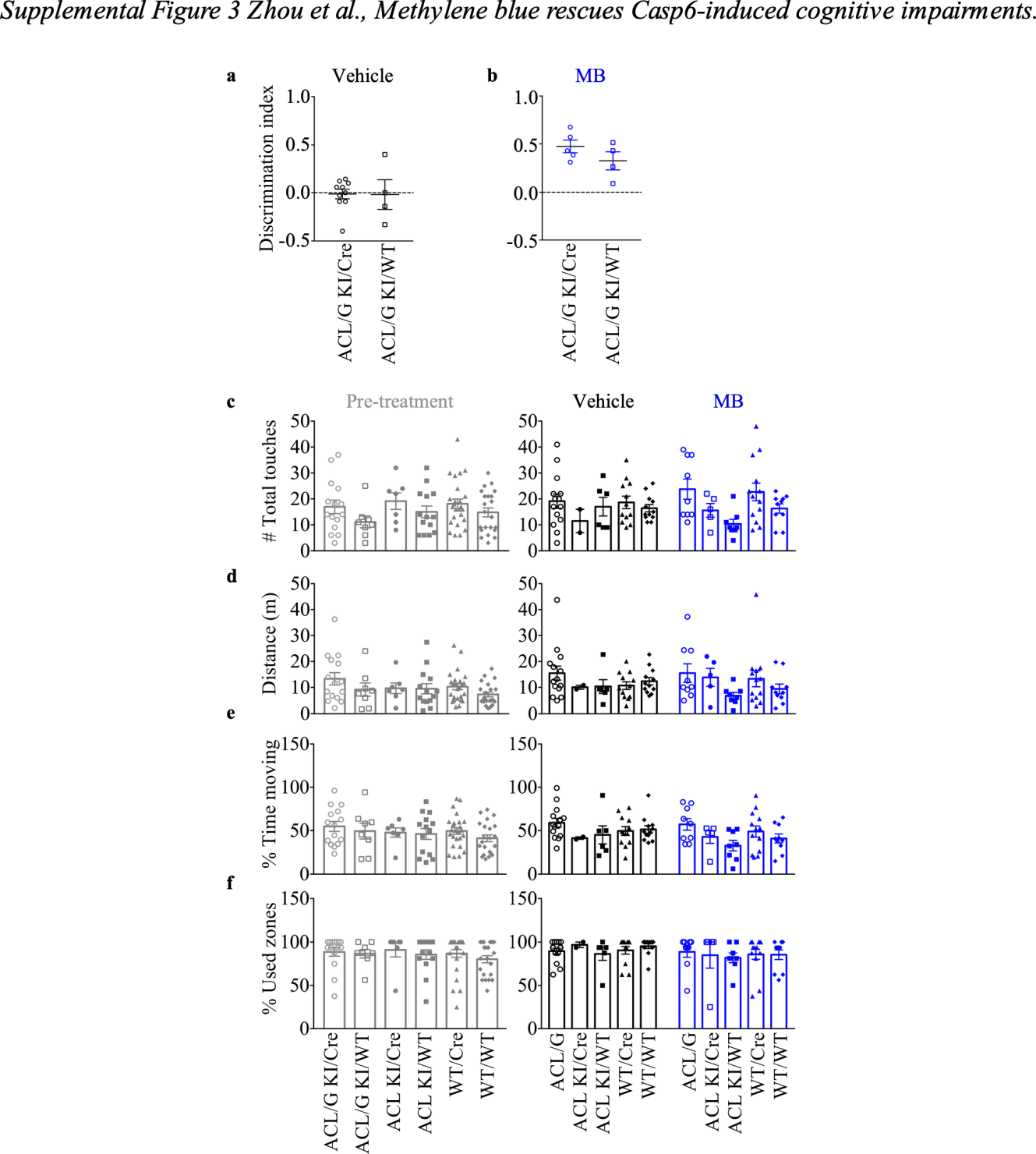


**Figure S3. NOR discrimination index and open-field locomotor ability between ACL/G or ACL KI/Cre and KI/WT.** **(a&b)** No statistical difference was shown in the discrimination index between vehicle-treated ACL/G KI/Cre and ACL/G KI/WT mice (**a**), or between MB-treated ACL/G KI/Cre and ACL/G KI/WT mice (**b**) by Student's t-test. **(c-f)** The total numbers of touches to both objects (**c**), the distance travelled (**d**), the % time moving (**e**), and the % used zones (**f**) before treatment (pre-treatment), and in vehicle- or MB-treated ACL/G (KI/Cre and KI/WT), ACL KI/Cre, ACL KI/WT, WT/Cre and WT/WT mice measured during NOR task. The total number of mice per group is indicated in Supplemental Figure S2. Each mouse tested is represented by one symbol. Bars represent the mean and error bars the s.e.m. No statistical difference was shown by one-way ANOVA.


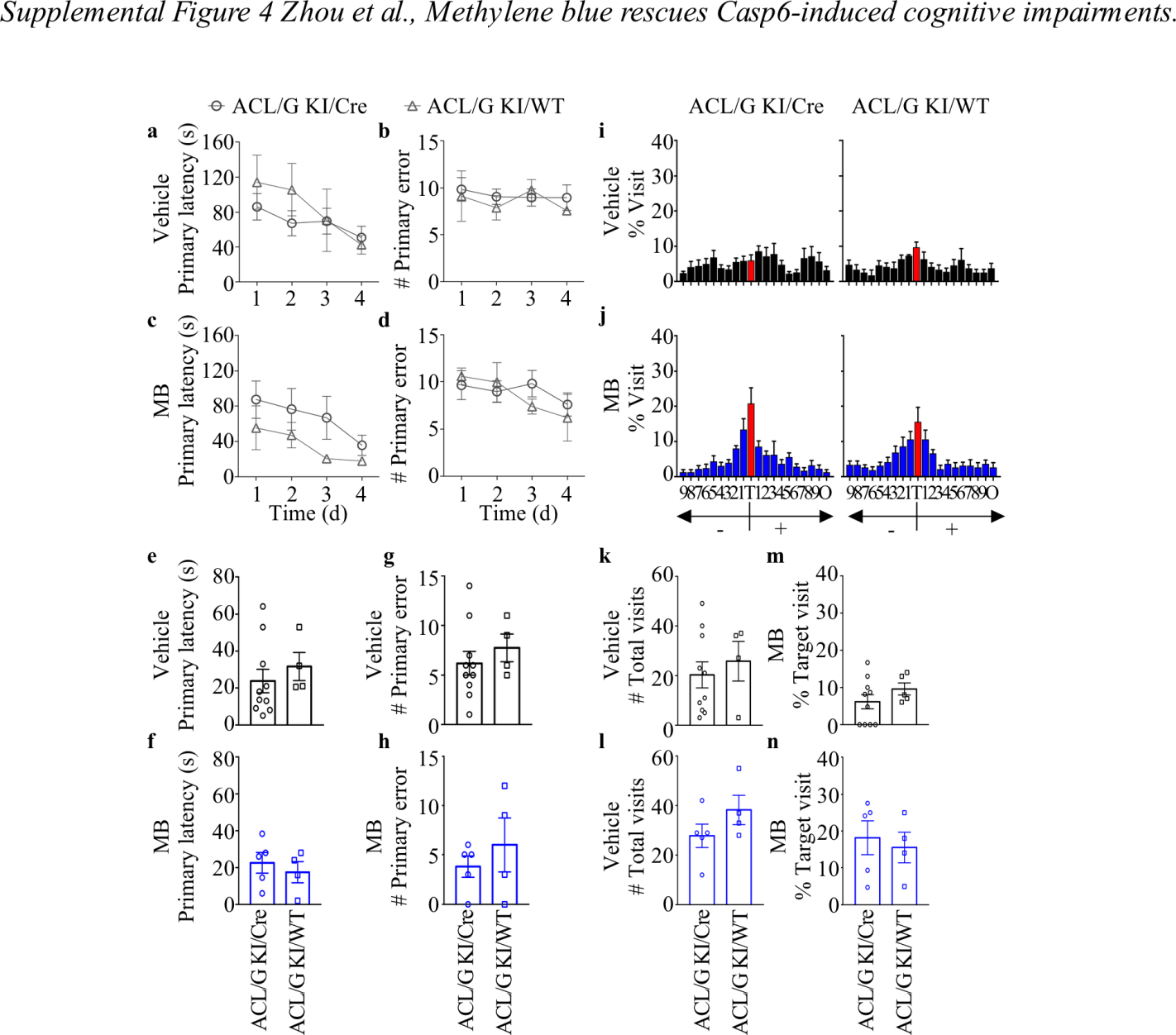


**Figure S4. No difference was seen between ACL/G KI/Cre and ACL/G KI/WT during Barnes maze.** **(a-d)** Primary latency (**a&c)** and primary errors (**b&d)** during spatial acquisition training in vehicle-treated ACL/G KI/Cre (n=10) and ACL/G KI/WT (n=4) **(a&b)**, and MB-treated ACL/G KI/Cre (n=5) and ACL/G KI/WT (n=4) (**c&d)** mice. No statistical difference was shown by repeated-measures two-way ANOVA. **(e-n)** Primary latency (**e&f)**, primary errors (**g&h)**, % visit to each hole **(i&j)**, number of total visits **(k&l),** and % visit to the target hole **(m&n)** during probe test in vehicle-treated **(e,g,k,l)**, and MB-treated (**f,h,m,n)** ACL/G KI/Cre and ACL/G KI/WT mice. Each mouse tested is represented by one symbol. Bars represent the mean and error bars the s.e.m. **(e-h,k-n)** No statistical difference was shown by Student’s test.


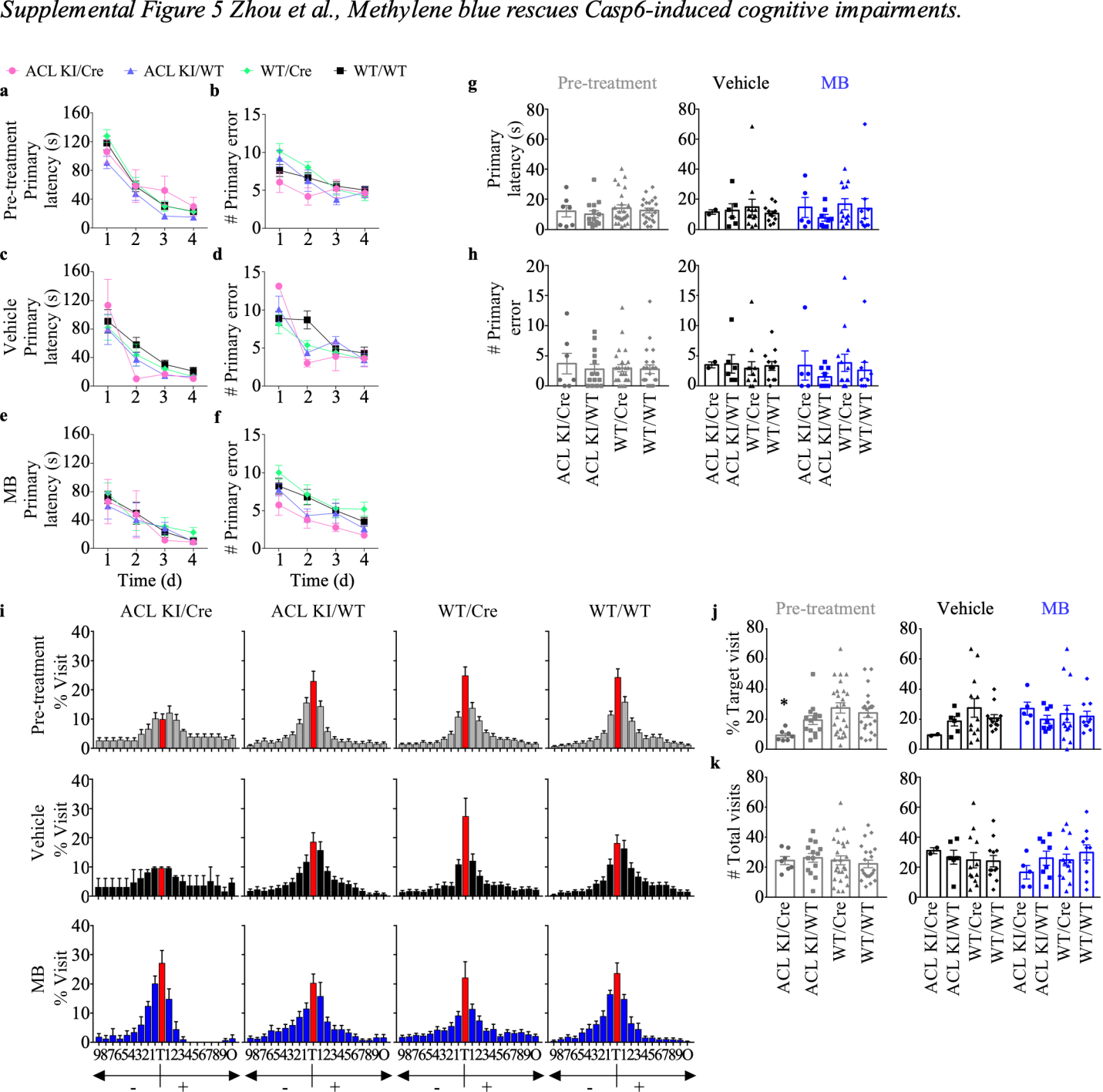


**Figure S5. MB reversed spatial memory impairments of ACL KI/Cre mice during Barnes maze.** **(a-f)** Primary latency (**a,c,e)** and primary errors (**b,d,f)** during spatial acquisition training in pre-treated ACL KI/Cre (n=7), ACL KI/WT (n=14), WT/Cre (n=25), and WT/WT (n=22) (**a&b),** vehicle-treated ACL KI/Cre (n=2), ACL KI/WT (n=6), WT/Cre (n=12), and WT/WT (n=12) **(c&d)**, and MB-treated ACL KI/Cre (n=5), ACL KI/WT (n=8), WT/Cre (n=13), and WT/WT (n=10) (**e&f)** mice. No statistical difference was shown with repeated-measures two-way ANOVA in **a,b**. **(g-k)** Probe test primary latency (**g)**, primary errors **(h),** % visit to each hole (**i)**, % visit to the target hole **(j)**, and number of total visits (**k**) were represented as mean and s.e.m. Each one symbol represents one individual mouse data. **(g,h,j,k)** Statistics were assessed with one-way ANOVA. Significance was observed only in the left panel of **j** (*p*=0.022). **p*<0.05 vs vehicle-treated WT/WT in Dunnett’s post-hoc analysis.

**
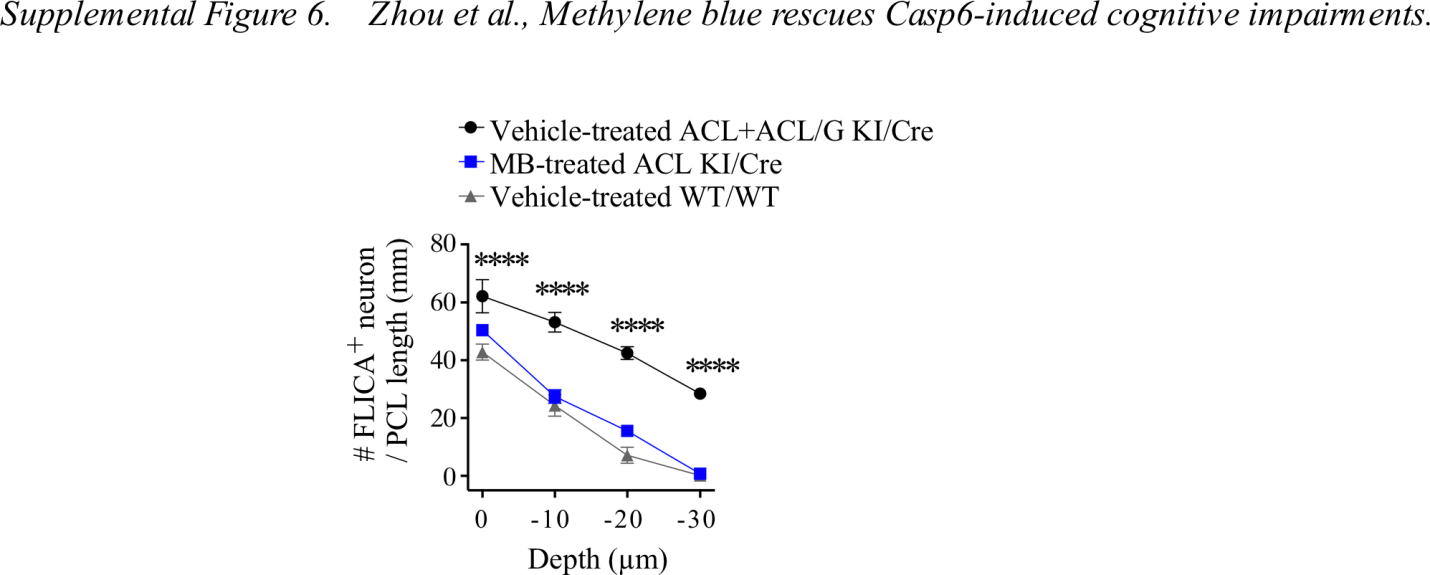
**

**Figure S6. FLICA-Casp6 activity in acute brain slice CA1 neurons.** Quantification of the numbers of FLICA-Casp6 positive neurons per µm along the pyramidal cell neurons (PCL) from the slice surface to -30-µm depth. Data represents mean ± s.e.m. Statistical evaluations were done with repeated-measures two-way ANOVA followed by Dunnett’s post-hoc analysis (depth *p*<0.0001, group *p*=0.0003, interaction *p*=0.0006). ***p*<0.01 vs vehicle-treated WT/WT.


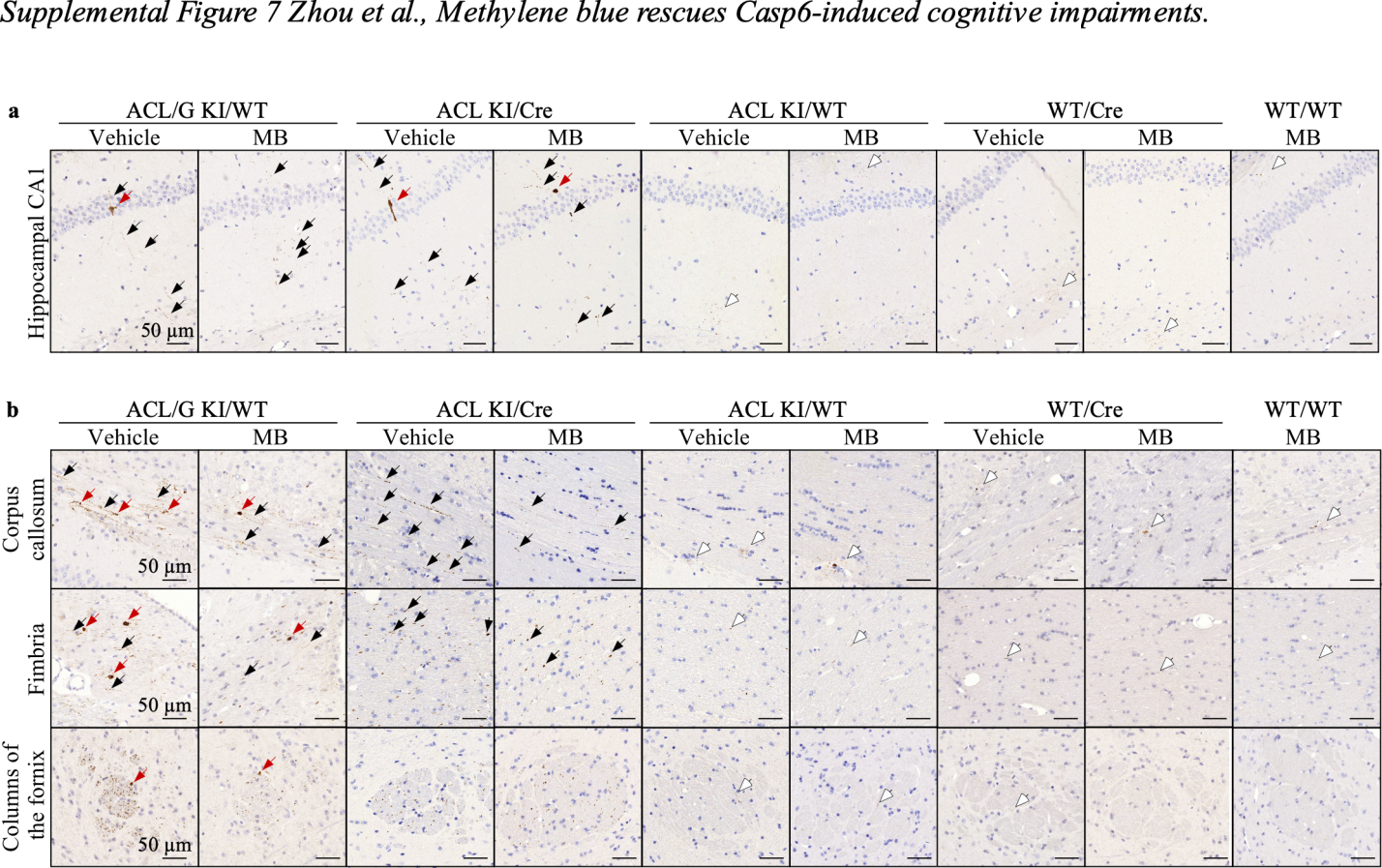


**Figure S7. Tub∆Casp6 immunostaining in hippocampal CA1 and fiber tracts. (a)** Representative micrographs of immunopositive Tub∆Casp6 in hippocampal CA1 regions of vehicle-treated ACL/G KI/WT (n=3), ACL KI/Cre (n=1), ACL KI/WT (n=3), WT/Cre (n=3), WT/WT (n=3) mice, and MB-treated ACL/G KI/WT (n=1), ACL KI/Cre (n=5), ACL KI/WT (n=3), WT/Cre (n=3), WT/WT (n=3) mice. Bar=50 µm. **(b)** Micrographs of immunopositive Tub∆Casp6 in the white matter of corpus callosum, fimbria, and the column of the fornix. Bar=50 µm. (**a&b)** Red arrow indicates immunopositive compact structures, black arrow indicates immunopositive neurites, and white arrow indicates punctate staining.

**
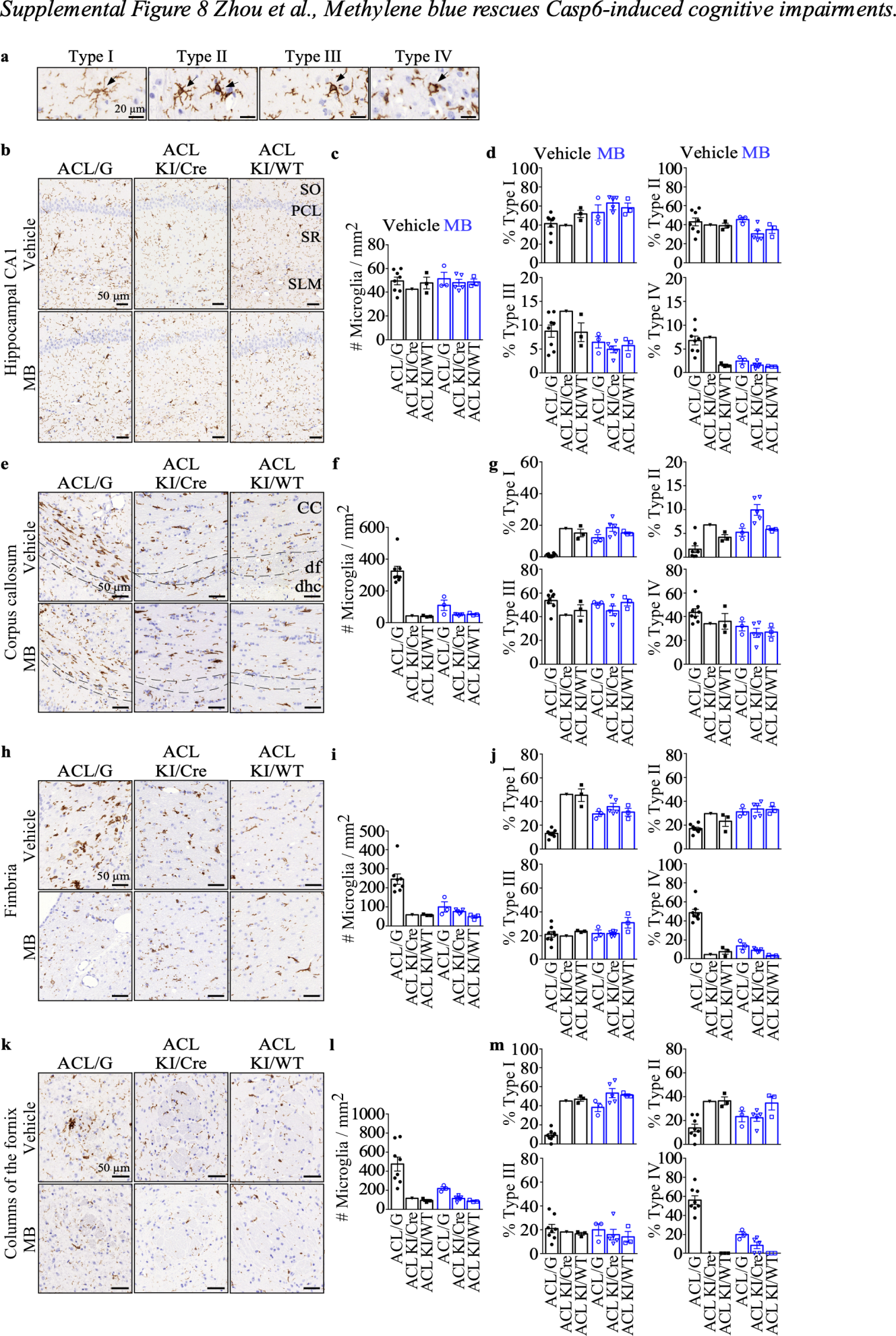
**

**Figure S8. Microglial activation in hippocampal CA1 region and fiber tracts.**

**(a)** Representative micrograph of subtype I, II, III, and IV microglia used for subtype classification. **(b-m)** Representative micrographs, Bar = 50µm (**b,e,h,k**), quantitation of numbers of Iba1-immunopositive microglia/mm^2^ (**c,f,i,l)** and subtype I-IV microglia (**d,g,j,m)** in hippocampus CA1 (**b-d)**, corpus callosum (**e-g)**, fimbria (**h-j)**, and fornix (**k-m)** of vehicle-treated ACL/G (KI/Cre n=5, KI/WT n=3), ACL KI/Cre (n=1), ACL KI/WT (n=3) or MB-treated ACL/G (KI/Cre n=2, KI/WT n=1), ACL KI/Cre (n=5), ACL KI/WT (n=3) mice. Data represents mean and s.e.m. Each symbol represents data from one mouse.


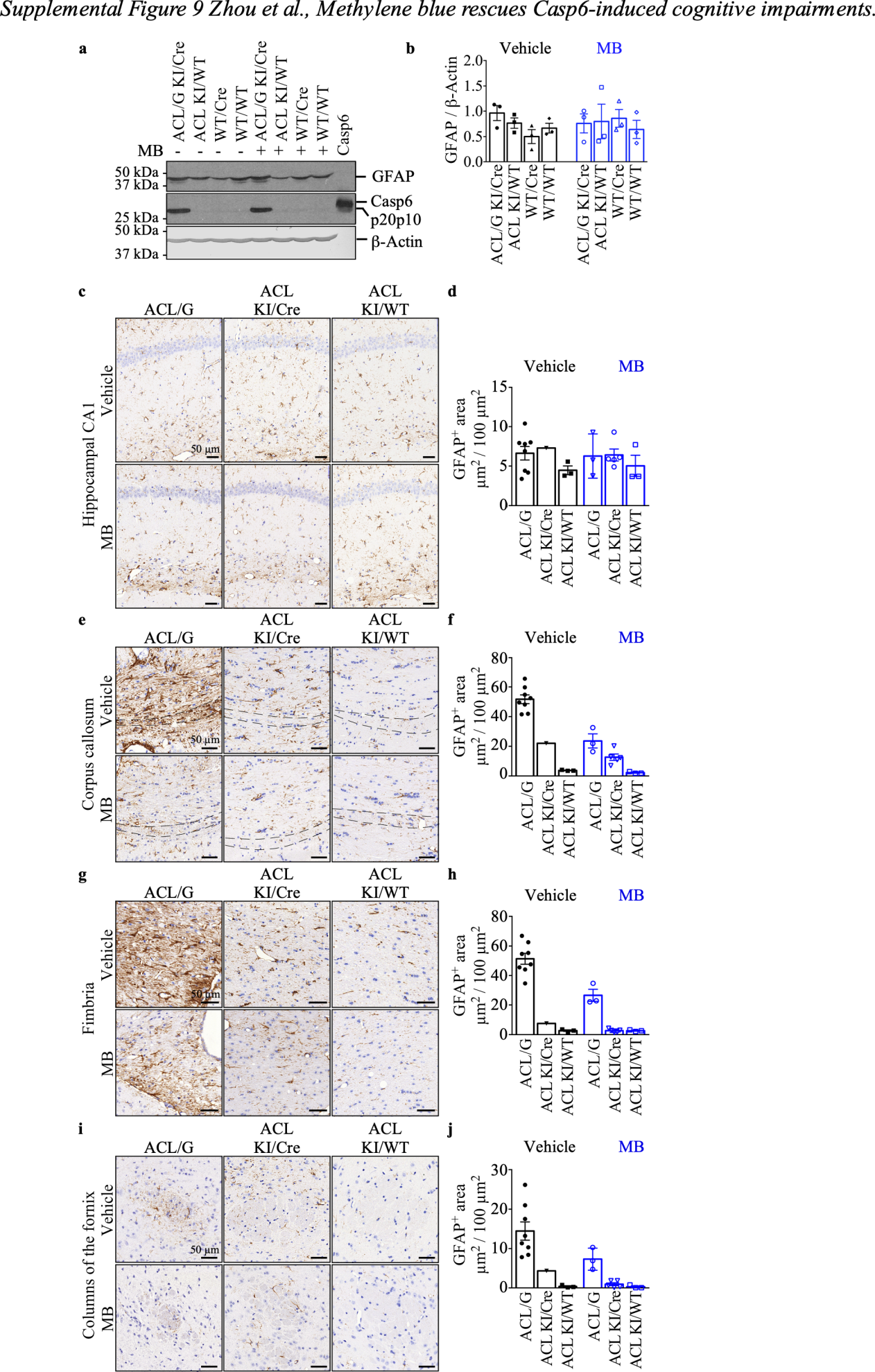


**Figure S9. Astroglial activation in Casp6-expressing brains.**

(**a)** Western blot of GFAP expression in vehicle- or MB-treated ACL/G KI/Cre, ACL KI/WT, WT/Cre and WT/WT hippocampus. (**b)** Quantification of GFAP over β-Actin. Data shown as mean and s.e.m. No statistical difference was shown by one-way ANOVA. (**c-j)** Representative micrographs, bar = 50 µm **(c,e,g,i**) and quantitation (**d,f,h,j)** of GFAP-immunopositive astrocytes (µm^2^ immunopositive GFAP/100µm^2^ area) in hippocampus CA1 (**c&d)**, corpus callosum (**e&f)**, fimbria (**g&h)**, and the columns of the fornix (**i&j)** of vehicle-treated ACL/G (KI/Cre n=5, KI/WT n=3), ACL KI/Cre (n=1), ACL KI/WT (n=3) or MB-treated ACL/G (KI/Cre n=2, KI/WT n=1), ACL KI/Cre (n=5), ACL KI/WT (n=3) mice. Data represents mean and s.e.m.
